# Activation and Role of Astrocytes in Ischemic Stroke

**DOI:** 10.3389/fncel.2021.755955

**Published:** 2021-11-17

**Authors:** Xin-Ya Shen, Zhen-Kun Gao, Yu Han, Mei Yuan, Yi-Sha Guo, Xia Bi

**Affiliations:** ^1^Graduate School of Shanghai University of Traditional Chinese Medicine, Shanghai, China; ^2^Department of Sport Rehabilitation, Shanghai University of Sport, Shanghai, China; ^3^Department of Rehabilitation Medicine, Shanghai University of Medicine and Health Sciences Affiliated Zhoupu Hospital, Shanghai, China

**Keywords:** astrocyte, oxidative stress, cerebral edema, glutamate, inflammation, blood–brain barrier, ischemic stroke

## Abstract

Ischemic stroke refers to the disorder of blood supply of local brain tissue caused by various reasons. It has high morbidity and mortality worldwide. Astrocytes are the most abundant glial cells in the central nervous system (CNS). They are responsible for the homeostasis, nutrition, and protection of the CNS and play an essential role in many nervous system diseases’ physiological and pathological processes. After stroke injury, astrocytes are activated and play a protective role through the heterogeneous and gradual changes of their gene expression, morphology, proliferation, and function, that is, reactive astrocytes. However, the position of reactive astrocytes has always been a controversial topic. Many studies have shown that reactive astrocytes are a double-edged sword with both beneficial and harmful effects. It is worth noting that their different spatial and temporal expression determines astrocytes’ various functions. Here, we comprehensively review the different roles and mechanisms of astrocytes after ischemic stroke. In addition, the intracellular mechanism of astrocyte activation has also been involved. More importantly, due to the complex cascade reaction and action mechanism after ischemic stroke, the role of astrocytes is still difficult to define. Still, there is no doubt that astrocytes are one of the critical factors mediating the deterioration or improvement of ischemic stroke.

## Introduction

Stroke is divided into two categories: ischemic and hemorrhagic, characterized by high incidence rate, high disability rate, and high mortality rate. Ischemic stroke is one of the leading causes of death and disability worldwide (Stapf and Mohr, 2002), accounting for approximately 70% of the total stroke cases (Feigin et al., 2015). A series of cellular and molecular-related events caused by the sudden disconnection of blood flow and subsequent reperfusion are the main causes of ischemic injury. The blood flow in the ischemic core area will be seriously reduced (McLeod et al., 2015). The insufficient supply of adenosine triphosphate (ATP) leads to the disorder of the brain environment and many cell death (Lipton, 1999). Unless blood flow recovery is performed within a few hours after ischemia, the death zone will gradually spread to the penumbra (Hossmann, 2012). At present, the clinical treatment of stroke is still dominated by conventional thrombolysis (Vidale and Agostoni, 2017). Still, most patients do not receive rapid and effective treatment because of the short time window, contraindications, etc. Therefore, the research on the treatment strategy of ischemic stroke has been a hot topic globally.

Astrocytes, also known as astroglia, are named for their unique star-shaped appearance. According to astrocytes’ morphology and spatial organization, they can be classically divided into three categories: fibrous astrocytes located in white matter, protoplasmic astrocytes in gray matter, and radial astrocytes surrounding ventricles (Swanson et al., 2004). In this review, we will ignore the morphological and functional diversity and complexity of different types of astrocytes and use an umbrella term, astrocytes. Astrocytes are the most abundant type of glial cells in the mammalian central nervous system (CNS) (outnumber neurons) and can affect all parenchymal cells due to contact and interaction with them. Under physiological conditions, astrocytes are provided with many functions.

On the one hand, astrocytes can regulate ion homeostasis and metabolism. On the other hand, it can also handle the formation of synapses (Ullian et al., 2001, 2004; Eroglu and Barres, 2010) and the energy supply of neurons. Besides, astrocytes also play an essential role in releasing neurotransmitters through exocytosis and ion channels (Sahlender et al., 2014). More importantly, astrocytes can regulate the flow of blood in the brain (Zonta et al., 2003; Mulligan and MacVicar, 2004; Attwell et al., 2010) and the blood–brain barrier (BBB) (Liebner et al., 2011). When ischemic stroke and other injuries occur, astrocytes are activated. In the early stage of ischemic injury, astrocytes will undergo significant morphological and functional changes, namely reactive gliosis. Notably, astrocytes will initiate a series of mechanisms to deal with body damage during ischemia. Animal experiments have shown that Glial fibrillary acidic protein (GFAP) null mice have more severe cerebral blood flow reduction and infarction volume increase than WT mice after middle cerebral artery occlusion (Nawashiro et al., 2000).

It is worth noting that astrocytes are also involved in the process of inflammation and neurotoxicity, so they also have a series of adverse effects. For example, glial scar induced infarct area expansion and axonal growth inhibition. These characteristics provide a basis for the idea that astrocytes are a therapeutic strategy for ischemic stroke. In this article, based on the most recent experimental data and research advances, the mechanisms of astrocyte activation have been elucidated, and several significant functions of astrocytes have been addressed holistically. To summarize, this article allows a comprehensive understanding of the role of astrocytes in ischemic stroke and sheds new light on astrocytes as a therapeutic strategy for ischemic stroke.

## The Changes of Astrocytes After Ischemia

Astrocytes are the most widely distributed type of cells in the mammalian brain, and also the largest type of glial cells. The number of astrocytes in the brain is about five times that of neurons. They continuously cover the entire CNS and can transmit information and maintain homeostasis (Sofroniew and Vinters, 2010). Astrocyte activation refers to the phenotypic transformation from resting state to reactive state (Sofroniew, 2009). The related changes of reactive astrocytes include hyperplasia (Li et al., 2014) and swelling (Swanson et al., 2004; Nedergaard and Dirnagl, 2005; Liu et al., 2014), and the increase of associated proteins such as GFAP, S100beta (Dagonnier et al., 2021), and vimentin. However, the peak time of GFAP in the serum of patients with brain injury is earlier, more specific and more sensitive (Senn et al., 2014). Therefore, the GFAP is considered a reliable marker of reactive astrocytes (Panickar and Norenberg, 2005). The proliferation of reactive astrocytes is also known as reactive gliosis. Reactive gliosis is a characteristic change in the morphology and function of astrocytes in many nervous system diseases (such as ischemic stroke, neurotrauma, and neurodegenerative diseases), which profoundly affects the process of disease progression and recovery. Many experiments have shown that it is closely related to the formation of glial scar (Sofroniew, 2015). However, reactive gliosis is not synonymous with scar formation, nor is it a consistent process. On the contrary, astrocyte proliferation is a delicate and gradual change of genes and cells.

**FIGURE 1:**
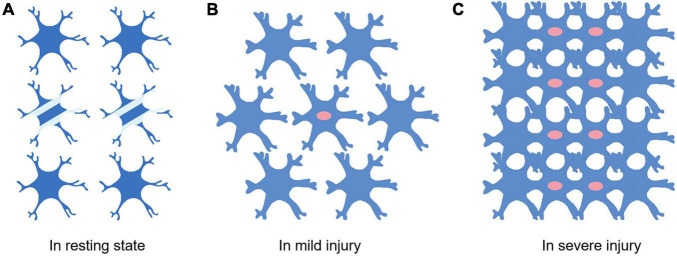
Differences of astrocytes in different extent of injury. **(A)** Astrocytes in the resting state. Not all astrocytes express detectable levels of GFAP (Two cells in the middle of **A**). There are clear and definite boundaries between cells. Little or no proliferation. **(B)** Most astrocytes express detectable levels of GFAP when the injury occurs. The astrocyte soma swells and proliferation begins to appear (The cells with red nuclei represent proliferating astrocytes). **(C)** Swollen astrocytes start proliferating massively. The boundaries between cells are destroyed.

Astrocytes vary with the severity of injury or distance from the lesion. Generally speaking, it is a dynamic process from swelling and proliferation to glial scar formation. Under physiological conditions, astrocytes cover the whole CNS in a continuous and almost non overlapping manner, and many astrocytes do not express detectable GFAP (Figure 1). When damage occurs and to a lesser extent, the expression of GFAP was upregulated and became detectable, and the cell bodies and processes of astrocytes became hypertrophic (Kajihara et al., 2001), but the boundaries of each astrocyte were clear and did not overlap. When the injury is serious, the expression of GFAP is significantly upregulated, the cell body of astrocytes are obviously hypertrophic, and a large number of proliferation and diffusion lead to the overlap between cells. Finally, proliferating astrocytes will form glial scars between damaged areas and healthy tissues, which can play different roles in the disease’s progress.

**FIGURE 2:**
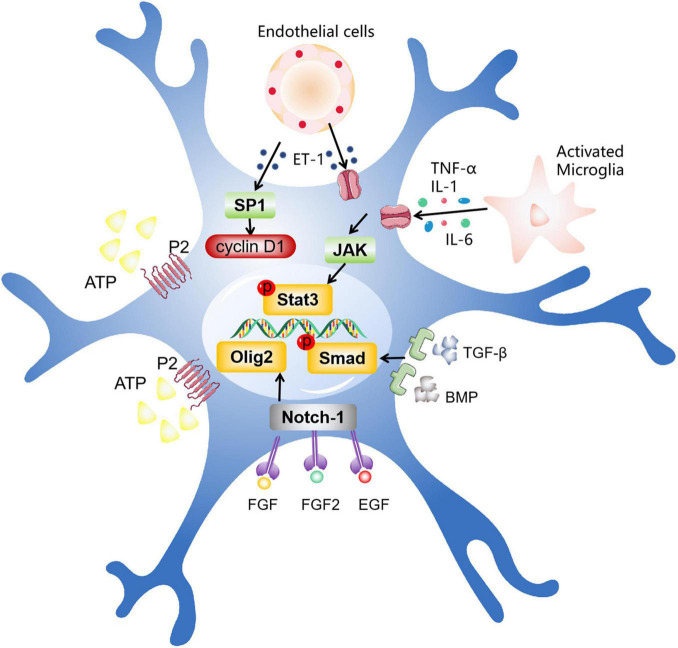
The intracellular mechanisms related to astrocyte activation. After ischemic stroke, different types of cells promote astrocyte activation by secreting various factors, including activated microglia, dead neurons, endothelial cells, and other cells. These factors act by entering the cell via multiple pathways, such as the JAK/STAT3 pathway, the Olig2 pathway, TGF-β/The Smad pathway, and other pathways.

Next, the intracellular mechanism of activation and proliferation of astrocytes is discussed. Numerous studies have shown that microglia, the first barrier of the immune system, are the first cells to sense damage and respond (Nakajima and Kohsaka, 2004). The activation of astrocytes may be related to some inflammatory factors released by microglia, such as transforming growth factor-alpha (TGF-α), interleukin-6 (IL-6), leukemia inhibitory factor (LIF), and tumor necrosis factor-α (TNF-α) (Balasingam et al., 1994; Winter et al., 1995; Klein et al., 1997; Rabchevsky et al., 1998). In addition, dead neurons and endothelial cells are also involved in the activation of astrocytes. They mainly release cytokines to regulate the activation and proliferation of astrocytes. Here, we mainly introduce several signaling pathways and transcription factors closely related to astrocyte activation (Figure 2).

### JAK/STAT3 Signaling Pathway

JAK/STAT3 is a key part of many signaling pathways regulating cell growth, differentiation, survival and pathogen resistance. This pathway involves gp130 receptor family, which helps regulate B cell differentiation, plasma cell generation and acute phase response. The related cytokines can bind to gp130 and activate Janus kinase (JAK), thus activating the pathway (Justicia et al., 2000; Xia et al., 2002). These cytokines are usually released by activated microglia, such as IL-1, IL-6, and TNF-α. A large number of studies have shown that the activation of astrocytes after ischemic stroke is closely related to JAK/STAT3 pathway (Ceyzériat et al., 2016). Neurotoxin mediated activation of STAT3 and increased expression of GFAP can be attenuated by inhibition of JAK/STAT3 signaling pathway (Sriram et al., 2004). Recent studies have demonstrated that JAK-STAT3 signaling pathway plays a crucial role in 17β-estradiol induced phenotypic transformation of reactive astrocytes. Exogenous 17β-estradiol administration completely rescued JAK/STAT3 pathway and astrocyte activation and proliferation (Wang et al., 2020). Consistent with previous results, reactive astrocyte proliferation and neuronal death induced by neurotoxicity are also regulated by JAK/STAT3 signaling pathway (O’Callaghan et al., 2014; Toral-Rios et al., 2020). These findings further confirm the vital role of JAK/STAT3 in inducing astrocyte activation and proliferation. Therefore, we can reasonably speculate that the classically activated microglia may activate this signaling pathway and finally cause the activation and proliferation of astrocytes (Buffo et al., 2010). Interestingly, recent studies have shown that ET-1 can also promote the proliferation of astrocytes after stroke through JAK/STAT3 signaling pathway (Cheng et al., 2019). The cytokines that activate this pathway need to be further explored and studied.

### Olig2

In CNS, the Olig2 transcription factor is widely existed, which plays a critical role in the cellular homeostasis of developing, mature and injured brain by involving various mechanisms (Ono et al., 2009). The expression of Olig2 in astrocytes can be seen in different injury models (Buffo et al., 2005). Previous animal experiments have shown that the emergence of Olig2 progenitor cells precedes reactive astrocytes, and Olig2 will migrate to the cytoplasm at the first time after injury (Magnus et al., 2007). Subsequent *in vitro* studies showed that the shift of Olig2 was achieved through the upregulation of Notch-1. This translocation may mediate the differentiation of astrocytes. Consistent with the previous conclusion, at least part of reactive astrocytes are derived from Olig2 progenitor cells (Tatsumi et al., 2008). In addition, the deletion of the cortical Olig2 gene can lead to the decrease of reactive astrocyte proliferation (Marshall et al., 2005; Cai et al., 2007; Chen et al., 2008). All these experiments show that Olig2 plays an irreplaceable role in the activation of astrocytes. However, there is no data to support the upstream mechanism of Olig2 expression. It is worth noting that experiments have proved that fibroblast growth factor (FGF) can induce the expression of Olig2 (Gabay et al., 2003). Similarly, FGF2/epidermal growth factor (EGF) can cause the expression of Olig2 and the differentiation of astrocytes (Dromard et al., 2007). These data provide a possibility that the activation of astrocytes induced by Olig2 may be regulated by FGF2, EGF, and other transcription factors.

### TGF-β/Smad Signaling Pathway

TGF-β/Smad signaling pathway signaling pathways are involved in many cellular processes, including cell growth, cell differentiation, apoptosis, cell homeostasis and other cellular functions (Budi et al., 2017). Transforming growth factor-β (TGF-β) and bone morphogenic protein (BMP) are two members of this signaling pathway. By stimulating their receptors, Smad family transcription factors in astrocytes can be activated. It has been proved that active BMP can activate Smad and promote the differentiation of astrocytes, and this effect can be eliminated by the inhibition of BMP (Fukuda et al., 2007). TGF-β can also regulate the differentiation of astrocytes (Sato, 2006; Sun et al., 2015; Li et al., 2017). Recent experiments have reached a similar conclusion that the continuous activation of signal pathway leads to the formation of abnormal glial scar, which can be reversed by the inhibition of this signaling pathway (Moon and Fawcett, 2001; Zhang et al., 2020). In addition, BMP can regulate cell composition by controlling the differentiation of neural stem cells, and ultimately regulate the phenotypic transformation of astrocytes (Xiao et al., 2010). In particular, chondroitin sulfate proteoglycan (CSPG), which is a component of extracellular matrix (ECM) of glial scar, is generally believed to hinder the growth of axons. By inhibiting TGF-β, the expression of glial scar and CSPG decreased significantly (Logan et al., 1994), which provides evidence for the involvement of TGF-β in the activation and proliferation of astrocytes (Susarla et al., 2011).

### Other Ways

Adenosine triphosphate (ATP) is an important mediator of communication between neurons and glial cells, and plays an important role in maintaining the normal function of the CNS (Pascual et al., 2005; Lee et al., 2013). It has been shown that ATP can bind with P2 Purinergic receptor, activate STAT3 in astrocytes, and regulate the activation and function of astrocytes (Neary et al., 2005). The application of inhibitors to P2 Purinergic receptor can reduce the expression of STAT3 (Washburn and Neary, 2006). Furthermore, as a transcription factor induced by oxidative stress in cortical neurons, the specificity protein 1 (SP1) increases its activity after the damage of the CNS and regulates the survival of neurons (Ryu et al., 2003). Cyclin D1, the target gene of SP1, was found to regulate the activation of astrocytes (Zhu et al., 2007). The expression of cyclin D1 can be observed in reactive astrocytes after cerebral ischemia (Di Giovanni et al., 2005; Zhu et al., 2007). Interestingly, some studies have found that endothelin-1 (ET-1) can also induce astrocyte activation after brain injury (Koyama and Michinaga, 2012; Koyama, 2021). After the intervention with ET-1, the phosphorylation of SP1 and the activity of Cyclin D1 were increased. In addition, the proliferation of astrocytes induced by ET-1 would be reversed after the use of SP1 inhibitor (Michinaga et al., 2013). These evidences suggest that the activation and proliferation of astrocytes after brain damage are regulated by multiple pathways. Some other approaches and their specific mechanisms need to be studied in the future.

## The Role of Activated Astrocyte in Ischemic Stroke

Astrocytes are activated after ischemic stroke and become reactive astrocytes. Reactive astrocytes often play different roles according to the type, degree, location of ischemia and different time points after injury. At present, there are many studies on the role of astrocytes after ischemic stroke. It is undeniable that the response of astrocytes to injury is designed to protect the nervous system, although there are some accompanying side effects. However, its function is still controversial. Next, we discuss several controversial functions of astrocytes after ischemic stroke.

### The Positive Roles of Activated Astrocyte in Ischemic Stroke

As a mechanism to deal with the injury, reactive astrocytes have been proved to play a crucial role in many aspects of injury recovery. The discussion of the specific mechanism of these positive effects will help us reasonably develop new treatment strategies.

#### Alleviating Oxidative Stress

Under normal physiological conditions, reactive oxygen species (ROS) are produced in the brain, which can be eliminated by free radical scavengers such as catalase in cells to maintain the balance of free radicals in the body. However, under pathological conditions such as cerebral ischemia, reperfusion leads to the production of ROS (Mason et al., 2000). Then the homeostasis of ROS is broken, resulting in many free radicals accumulated in the body and then damage cell structure and human tissue, namely oxidative stress. ROS can damage nerve cells and vascular endothelial cells in a dangerous state. They activate the matrix metalloproteinase (MMP) system and lead to ECM degradation (del Zoppo, 2009). Glutathione (GSH) is an important antioxidant and free radical scavenger (Dringen, 2000). It participates in the redox reaction *in vivo* and can combine with peroxide and free radicals to reduce ROS toxicity (Dringen et al., 2015). Endogenous brain GSH is a crucial determinant to prevent the aggravation of ischemic injury (Mizui et al., 1992). Astrocytes are rich in GSH and GSH metabolism-related enzymes, which play a critical role in reducing oxidative stress toxicity (Griffin et al., 2005). Astrocytes can provide GSH directly to neurons and store nitric oxide (NO) into nitrosoglutathione in the cytoplasm, thus alleviating the oxidative stress injury of neurons. It has been proved that neurons co-cultured with astrocytes have a higher survival rate when injured by NO or hydrogen peroxide (Tanaka et al., 1999; Chen et al., 2001). In addition, neurons can synthesize GSH through astrocyte-derived cysteine (Wang and Cynader, 2000). Therefore, astrocytes can protect neurons from oxidative stress by synthesizing and releasing GSH. Experimental studies show that pyruvate can induce glial cells to upregulate GSH synthesis, thereby saving neurological function (Miao et al., 2011). In the animal model of ischemic stroke, intravenous dehydroascorbic acid can quickly enter the brain and convert into protective ascorbic acid in the brain, regulating ROS by upregulating GSH and other mechanisms (Spector, 2016).

More importantly, astrocytes themselves also have a set of antioxidant systems to cope with excessive ROS. First, astrocytes can convert oxidized GSH to reduced GSH via the pentose-phosphate pathway, thereby exerting the powerful antioxidant capacity of GSH. Moreover, Kelch-like enoyl-CoA hydratase-associated protein 1/nuclear factor erythroid two p45 subunit-related factor 2/antioxidant response element (Keap1/Nrf2/ARE) pathway has also been implicated in the regulation of oxidative stress in astrocytes. Under physiological conditions, Nrf2 binds to its adaptor protein Keap1 to form a heterodimer. The Keap1/Nrf2 complex is continuously degraded by ubiquitination, so the transcriptional activity of Nrf2 is inhibited. When ischemia occurs, the stimulation of ROS allows dissociation of the Keap1/Nrf2 complex, Nrf2 is released and translocates to the nucleus, where it binds to the ARE to regulate the transcriptional activity of antioxidant enzymes (Takahashi, 2021). The sulforaphane (classical Nrf2 activator) significantly increased GSH in astrocytes cultured *in vitro* (Takahashi et al., 2012).

Similarly, another pathway of astrocyte antioxidant capacity was found that adenosine monophosphate-activated kinase (AMPK) selectively regulates the expression of GCLM (glutamate-cysteine ligase regulatory subunit) in astrocytes via peroxisome proliferator-activated receptor-gamma coactivator-1α (PGC-1α) activation, which elevates GSH synthesis (Guo et al., 2018). However, the protective response brought about by such damage is short-lived and easily broken. This is also why, under the premise of possessing such a protective mechanism, the organism is still receiving further damage. Nevertheless, the inhibition or activation of relevant pathways can still be considered a therapeutic strategy.

#### Releasing Neurotrophic Factors

Neurotrophic factors are proteins that play an essential role in the development, survival and apoptosis of neurons. Astrocytes can release a variety of neurotrophic factors under normal physiological conditions, such as brain-derived neurotrophic factor (BDNF), nerve growth factor (NGF), glial cell line-derived neurotrophic factor (GDNF), ciliary neurotrophic factor (CNTF) and others (Ridet et al., 1997). Their expression will be increased after ischemia, which provides the nutrients needed for axon regeneration, promoting axon growth and nerve regeneration (Tokita et al., 2001). BDNF binds to high-affinity TrkB receptors and low-affinity p75NTR receptors to activate downstream signaling pathways, and plays a role in neuronal survival and apoptosis. Interestingly, astrocytes can respond to excessive BDNF signaling by secreting toxic NO (Colombo et al., 2012). In general, astrocyte-derived BDNF is beneficial to axonal myelination and neuronal function. Relevant experiments have proved that NGF is involved in the survival of neurons after early ischemic stroke (Lee et al., 1996).

What’s more, astrocyte-derived GDNF has been shown to have a positive effect on tight junction function and BBB permeability during neuroinflammation *in vitro* (Igarashi et al., 1999). CNTF is a single neurotrophic factor family and has been widely studied as an inducer of neuronal differentiation, survival and neurite growth. Accumulating experiments have proved that astrocyte-derived CNTF mediates neurogenesis in the subventricular areas after ischemic stroke (Kang et al., 2013; Jia et al., 2018). Besides, upregulation of Mesencephalic Astrocyte-Derived Neurotrophic Factor (MANF) and Cerebral Dopamine Neurotrophic Factor (CDNF) in astrocytes was observed in the experimental model of stroke, which alleviates the damage of endoplasmic reticulum stress (Shen et al., 2012; Cheng et al., 2013; Zhao et al., 2013). Given the positive effects of these neurotrophic factors after ischemic stroke, it is reasonable to enhance their expression by pharmacological treatment further to attenuate ischemic injury. Galectin-1 can regulate the proliferation of many cell types and plays a vital role after nervous system injury. Previous studies have shown that Galectin-1 can significantly enhance astrocytic BDNF expression and secretion, and that Galectin-1 intervention can reduce neuronal apoptosis and promote functional recovery (Qu et al., 2010). In addition, dexmedetomidine (Dex), an adrenergic receptor agonist, is commonly used as a drug for sedation and analgesia in pediatrics. An oxygen-glucose deprivation experimental model demonstrated that Dex could promote the expression of GDNF in astrocytes, thereby alleviating neurotoxicity (Yan et al., 2011).

#### Reducing Cerebral Edema

Cerebral edema after ischemic stroke is mainly divided into cytotoxic edema and angiogenic edema. In the early stage of ischemia, with hypoxia and lack of energy, a large amount of Na^+^ and H_2_O flow into the cells, resulting in cell swelling. With the development of cerebral ischemia, the BBB ruptures and plasma proteins leak into the extracellular space, resulting in angiogenic edema (Simard et al., 2007). Meanwhile, both neurotransmitter release and inflammatory responses can aggravate cerebral edema. Astrocytes are closely associated with the cerebral vasculature, and their critical role in the formation and clearance of brain edema has gradually been emphasized with the discovery of aquaporin-4 (AQP4). The AQP family regulates bidirectional water transport, and they share standard structural features: 6 transmembrane domains with intracellular carboxyl (c) and amino (n) termini (Ho et al., 2009). Among the known aquaporins, only aquaporin-1, AQP4, and aquaporin-9 are highly expressed in the CNS (Stokum et al., 2015). Among them, AQP4 localized to astrocyte endfeet is an extremely critical regulator of cerebral edema, which can immediately regulate the influx or efflux of water according to the osmotic gradient, thereby maintaining brain water content at an equilibrium.

Interestingly, the current study shows conflicting roles for AQP4 in post-ischemic cerebral edema. In several studies, knockout of the AQP4 gene was shown to reduce or prevent post-ischemic cerebral edema (Da and Verkman, 2004; Zhang et al., 2019). Consistent with the previous conclusions, the same results can be obtained by inhibiting the expression of AQP4 by different means (Cheng et al., 2018; Hao et al., 2021; Shi et al., 2021). These studies seem to suggest that AQP4 has a promoting effect on brain edema. However, this is because AQP4 is a key factor in regulating brain edema. Although AQP4 gene knockout improves brain edema, it also extinguishes the possibility of AQP participating in edema elimination, thus bringing a series of associated pathological injuries. Some studies support this view, AQP4 deficient mice show larger infarct volume and worse neurological function score after ischemia-reperfusion (Zeng et al., 2012). Similarly, AQP4 knockout mice had worse brain edema and clinical manifestations, including cortical freezing injury, brain abscess, brain tumor and hydrocephalus in the angiogenic edema model (Verkman et al., 2006; Papadopoulos and Verkman, 2007). In conclusion, AQP4 mediated astrocytes play an important role in improving cerebral edema after ischemic stroke. The unique advantage of AQP4 in regulating water transport determines its crucial position in cerebral edema. Its specific role should be judged according to different time and state after ischemia. The inhibition of AQP4 in the formation stage of edema and the activation in the elimination stage of edema will better reduce cerebral edema (Clément et al., 2020).

#### Protecting Neurons and Reducing Infarct Volume

Erythropoietin (EPO), initially found in fetal liver and adult kidney, can stimulate the production of red blood cells (Sasaki, 2003). More and more evidences show that EPO also exists in the central nervous system. Astrocytes are the primary source of EPO in the brain and can promote the Production of EPO during ischemia. The EPO interacts with erythropoietin receptor (EPOR) on neurons (Buemi et al., 2003), which also has a variety of functions, especially in the protection of the brain after ischemia (Nguyen et al., 2014; Mallet and Ryou, 2017). It was found that hypoxia inducible factor-1 (HIF-1) mediated the up regulation of Erythropoietin in the brain (Fandrey, 2004). Animal experiments have shown that hypoxia treatment for 1–6 h before middle cerebral artery occlusion can significantly reduce the infarct volume of mice. This is closely related to the upregulation of astrocyte derived EPO induced by HIF-1 (Bernaudin et al., 2002). Notably, the strong neuroprotective effect of Erythropoietin exists in all stages of ischemia. Administration of Erythropoietin 24 h before, during or 3 h after middle cerebral artery occlusion can reduce neuronal apoptosis and decrease the infarct volume by almost 75%. Even administration of Erythropoietin 6 h after occlusion has a partial protective effect (Brines, 2002; Larpthaveesarp et al., 2016). Consistent with previous conclusions, EPO treatment after cerebral ischemia can significantly reduce neuronal apoptosis and infarct volume in rats (Sirén et al., 2001; Zhao et al., 2015). Moreover, the experimental model *in vitro* showed that EPO secreted by astrocytes could activate EPOR on neurons and inhibit ischemia-induced neuronal apoptosis (Ruscher et al., 2002). The strong ability of astrocyte derived EPO is related to its close involvement in the cascade reaction after ischemic stroke, including reducing excitatory toxicity, oxidative stress and inflammatory response. In addition, EPO can also promote angiogenesis and maintain the integrity of BBB (Larpthaveesarp et al., 2016).

### The Negative Roles of Activated Astrocyte in Ischemic Stroke

Although reactive astrocytes have many positive effects, they are usually unstable and even reversed in some cases. These changes are closely related to the progress of the disease. At least some of them occur when there is severe ischemia and hypoxia.

#### Induction of Excitotoxicity

When cerebral ischemia occurs, excitatory amino acids will be over released. The Glutamate (Glu) predominant excitotoxicity is a major cause of neuronal death after ischemic stroke (Choi, 1988). Glu uptake is a major function of astrocytes in the early stage of cerebral ischemia. This function is mainly realized by Na^+^-dependent glutamate transporters, which are located in astrocytes (Huang and Bergles, 2004) and originally cloned from rat brain (Ullensvang et al., 1997). Glutamate aspartate transporter (GLAST) and glutamate transporter-1 (GLT-1, also known as EAAT2) can reduce excitotoxicity by ingesting glutamate from extracellular space (Anderson and Swanson, 2000). *In vitro* experiments have demonstrated that glutamate concentrations need to increase nearly one hundred-fold to be neurotoxic in the presence of a large number of astrocytes (Rosenberg and Aizenman, 1989). This illustrates the robust capacity of astrocytes to take up glutamate. However, glutamate uptake by astrocytes requires consuming a large amount of energy, which is challenging to maintain during ischemic stroke. Therefore, the glutamate uptake capacity of astrocytes is often impaired or even reversed when the ischemic stroke is severe (Longuemare and Swanson, 1995; Leonova et al., 2001; Pajarillo et al., 2019). The pathway by which astrocytes release glutamate is primarily the reversal of glutamate transporters. Excessive glutamate will lead to elevated intracellular Na^+^ and Ca^+^, and a massive influx of Ca^+^ will lead to mitochondrial dysfunction, protease activation, accumulation of ROS and NO release, ultimately causing excitotoxicity and neuronal death (Kostandy, 2012).

Astrocyte glutamate transporter is dependent on Na^+^, and the destruction of Na^+^/K^+^ – ATPase (NKA) after ischemia leads to Na^+^ imbalance. Therefore, the operation mode of the transporter is reversed, resulting in a large amount of Glu release (Camacho and Massieu, 2006). Consistent with the previous conclusion, the imbalance of NKA caused by high extracellular concentrations of K^+^ also resulted in the reversal of glutamate uptake (Longuemare et al., 1999).

Likewise, the cystine-Glu transporter is one of the pathways for Glu release, which acts by importing cystine in exchange for Glu, and most cystine glutamate transporters are localized in glial cells of the brain (Pow, 2001). In addition, the purinergic P2X_7_ ion channel may provide another way for astrocytes to release Glu (Duan et al., 2003; Malarkey and Parpura, 2008). P2X receptors are cation selective ion channels that show amplified responses in low external divalent cation solutions. It can be detected in astrocytes *in vitro* and may be upregulated after injury (Franke et al., 2001). Besides, anion channels associated with cell swelling can also release Glu. When ischemia occurs, most cells will experience swelling. With the emergence of low permeability, they can cope with this increase in volume by opening volume-regulated anion channels. A large number of experimental results strongly show that the anion channel is one of the main contributors to the release of glutamate from cultured astrocytes (Kimelberg et al., 1990; Osei-Owusu et al., 2018). Interestingly, increasing evidence suggests that the hemichannels composed of connexin43 on the astrocyte membrane also mediates the release of Glu (Ye et al., 2003; Parpura et al., 2004; Abudara et al., 2018; Xing et al., 2019). Astrocytes using hemichannels inhibitors or from connexin43 knockout mice showed reduced Glu release (Ye et al., 2003; Spray et al., 2006). Although multiple mechanisms of glutamate release have been discovered, the contribution and timing of action remain to be investigated. However, excessive glutamate release after injury will certainly cause excitotoxicity.

#### Inducing an Excessive Inflammatory Response

Astrocytes are one of the important regulators of the immune response of the CNS. With the development of brain injury after ischemia, astrocytes can aggravate the inflammatory response by releasing a large number of inflammatory factors, promote the activation and infiltration of other inflammatory cells, and trigger a cascade of inflammatory response. There is already substantial evidence that microglia are major players in initiating inflammatory responses, but multiple cell populations including astrocytes also contribute to these changes (Buffo et al., 2010; Pál et al., 2012). Previous animal experiments have shown that a large amount of leaked adenosine 5’-triphosphate (ATP) after ischemia can activate P2Y1 receptor on astrocytes, then promote the production of proinflammatory cytokines through nuclear factor-kappa B (NF-κB) pathway, and finally aggravate the inflammatory response (Kuboyama et al., 2011). Cerebral ischemia can induce astrocytes to produce a large number of inflammatory factors, including IL-1, IL-4, IL-6, and TNF-α, which are involved in the occurrence and development of inflammatory response (Pál et al., 2012; Ruscher et al., 2012). The expression of IL-1 was upregulated after cerebral ischemia, especially IL-1β, which is a crucial factor involved in the inflammatory response, which can further activate microglia and aggravate ischemic injury (Liu and Chopp, 2016). Although many studies have shown that astrocytes can also reduce the inflammatory response, the stimulation from the microenvironment in neuroinflammation may change astrocytes’ activity from beneficial to harmful neural tissue (Colombo and Farina, 2016). It is worth noting that recent studies have found that reactive astrocytes have two subtypes: A1 and A2. A1 is induced by inflammatory factors secreted by classically activated microglia, and it completely loses its original protective function and becomes a pro-inflammatory state, which can quickly lead to neuronal death in various ways (Liddelow et al., 2017).

### The Dual Role of Activated Astrocyte in Ischemic Stroke

Although the specific role of astrocytes is still controversial, there is no doubt that reactive astrocytes play an obvious dual role in some aspects, which is commonly known as the double-edged sword effect.

#### Astrocytes and Blood–Brain Barrier

**FIGURE 3:**
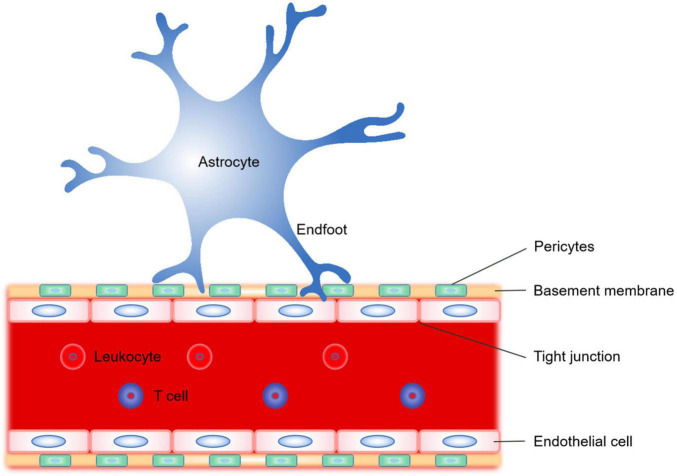
Schematic representation of the significant constituent structures of the blood–brain barrier (BBB). The BBB is a complex composed of astrocyte endfoot, vascular endothelial cells, tight junction, basement membrane, and pericytes. The astrocytes regulate the homeostasis and function of BBB mainly by transporting different derived factors through their endfoot.

The BBB is composed of astrocytes, vascular endothelial cells, tight junction, basement membrane, pericytes and T cells (Wevers and de Vries, 2016; Figure 3). BBB is a multicellular vascular structure that separates CNS from peripheral blood circulation. BBB mainly plays a barrier function, strictly controls the passage of molecules and ions, and protects the brain from the invasion of pathogens (Obermeier et al., 2013). The destruction of BBB after ischemic stroke (Arba et al., 2017) will lead to a series of pathological changes, such as vascular edema, the opening of tight junctions between endothelial cells, leukocyte infiltration and toxic molecules invading the brain. The role of astrocytes in the BBB in different physiological and pathological states has long been concerned (Abbott, 2002; Abbott et al., 2006). Astrocytes regulate the homeostasis and function of BBB mainly through derived factors (Figure 3).

In fact, it has been proved that astrocytes can regulate the permeability of BBB by secreting different factors. For example, astrocytes can secrete vascular endothelial growth factor (VEGF) to increase the permeability of BBB (Jiang et al., 2014a) and eventually lead to leukocyte extravasation when it is exposed to the pro-inflammatory cytokine IL-1β (Argaw et al., 2009, 2012). The VEGF mediated injury is likely caused by the downregulation of tight junction associated proteins in endothelial cells (Argaw et al., 2012). However, late administration of VEGF can significantly enhance the promotion of angiogenesis and improve neurological function during stroke recovery (Zhang et al., 2000). In addition, Astrocytes can release MMP to destroy endothelial tight junction associated proteins and some extracellular matrix molecules. Animal experiments of cerebral ischemia have shown that MMP make BBB open by degrading endothelial tight junction associated proteins, and MMP inhibitors can prevent this result (Asahi et al., 2001; Yang et al., 2007; Yang and Rosenberg, 2011; Zhang et al., 2018). Actually, astrocytes can produce NO to increase the permeability of BBB, which may be achieved through cyclic guanosine monophosphate pathway (Gu et al., 2012; Fu et al., 2014; Jiang et al., 2014b). The toxic damage caused by excitatory substances such as glutamate (Glu) is one of the important mechanisms of opening of BBB. The Glu released by astrocytes activates N-methyl-D-aspartate (NMDA) receptors on endothelial cells, thereby inducing vasodilation and increasing the permeability of BBB (Sharp et al., 2003; András et al., 2007; Liu et al., 2010; Lu et al., 2019). ET-1 is a member of endothelin family and is considered to be an endogenous long-acting vasoconstrictor. Similarly, astrocytes in cerebral ischemia model can produce ET-1. Overexpression of ET-1 can increase the permeability of BBB and aggravate brain injury (Lo et al., 2005; Hung et al., 2015).

Astrocytes can also produce some protective factors to maintain the normal function of BBB. Angiopoietin-1 (Ang-1) can inhibit endothelial cell apoptosis and reduce vascular atrophy and degeneration. Studies have shown that astrocytes can produce Ang-1 and protect BBB by increasing the expression of endothelial tight junction protein (Lee et al., 2003; Yu et al., 2012). Sonic hedgehog (SHH) is a glycoprotein mainly produced by astrocytes, endothelial cells and immune cells (Hill et al., 2021). It can protect endothelial cells and promote angiogenesis (Liu et al., 2018). The animal experiment of permanent middle cerebral artery occlusion (pMCAO) model shows that SHH can up regulate the expression of Ang-1, so as to reduce brain edema and maintain the permeability of BBB (Xia et al., 2013). In addition, Insulin-Like Growth Factor-1 (IGF-1) has been shown to promote cell proliferation and differentiation, protect neurons, and is mainly found in glial cells such as astrocytes (Pitt et al., 2017; Wrigley et al., 2017). Experimental studies have shown that IGF-1 can maintain the normal permeability of the BBB by stabilizing the microvascular cytoskeleton under ischemic conditions (Bake et al., 2019). Moreover, apolipoprotein E (APOE), as an important apolipoprotein in plasma, is mainly responsible for the transport, storage, and metabolism of various lipids (Marais, 2019). Astrocytes are a major source of APOE (Morikawa et al., 2005), and experiments have shown that loss of APOE decreases endothelial tight junction protein expression and thereby disrupts the BBB (Koyama, 2014). In addition to this, several studies have shown that astrocyte derived retinoic acid (RA) and glial derived neurotrophic factor (GDNF) are similarly involved in the maintenance of the normal function of the BBB through various mechanisms (Mizee et al., 2013; Xiao et al., 2014). BBB is a major difficulty in drug research and development, which excludes more than 98% of the small molecule neurotherapeutic drugs and almost all the large molecule drugs (Al-Ahmady, 2018). Astrocytes, as the key regulators of BBB permeability and the main participants of stress response after ischemic stroke, have been gradually recognized to play an irreplaceable role in the development of therapeutic strategies (Cohen and Dillin, 2008; Song et al., 2016).

#### Astrocytes and Ion Homeostasis

The maintenance of normal ion gradient between inside and outside cells is one of the necessary conditions to ensure cell survival and perform physiological processes. Brain metabolism is fast and lacks the glucose and oxygen needed reserves, so it is susceptible to ischemia. At the onset of ischemic stroke, a sudden decrease in cerebral blood flow leads to rapid depletion of the remaining energy source, and a severe energy shortage occurs in the ischemic core area, which will lead to the disruption of ion gradients, cell death and aggravating the ischemic injury. There are many kinds of ion channels in astrocytes, which can regulate the ion concentration inside and outside the cells, maintain the ion homeostasis and reduce the damage. Here, we mainly present the homeostatic regulation of Na^+^ and K^+^ mediated by Na^+^/K^+^-ATPase (NKA) and its underlying co-transporters.

In the resting state, astrocytes maintain ionic homeostasis with low Na^+^ and high K^+^, which is mainly achieved through the regulation of NKA. NKA is one of the most highly expressed proteins in astrocytes (Stoica et al., 2017). Thus, even in the resting state, inhibition of NKA leads to an immediate Na^+^ rise in cultured astrocytes (Rose and Ransom, 1996; Rose et al., 1998). Intracellular Na^+^ homeostasis is also dependent on intact energy metabolism as NKA requires ATP for function (Gerkau et al., 2017). At the time of ischemic stroke, significant Na^+^ elevations occur in the area of injury (Rossi et al., 2000). The large increase of intracellular Na^+^ may lead to the abnormality of the glutamate transporter, which will promote the excessive accumulation of Glu, resulting in excitotoxicity and cell death (Szatkowski and Attwell, 1994). When the intracellular Na^+^ is abnormally increased, the regulation of NKA is the main pathway but not the only one. Other Na^+^ associated transporters are involved, such as Na^+^/Ca2^+^ exchanger (NCX), Na^+^-HCO_3_^–^ co-transporter (NBC), sodium-coupled neutral amino acid transporters 3 and 5 (SNAT3/5) and gamma-aminobutyric acid transporter (GAT). Their normal working mode is Na^+^ influx, however, this pattern reverses in accordance with ion gradients and membrane potential. Thus, they also emerged as regulators of Na^+^ efflux. NCX can exchange 3 Na^+^ for 1 Ca^2+^ and also mediates Na^+^ influx and efflux (Verkhratsky et al., 2018; Rose et al., 2020). Another transporter with reversal potential is NBC, which can regulate HCO_3_^–^ in astrocytes (Chesler, 2003). In addition, the reversal of the working mode of some transporters has been proved to be related to the uptake of transmitters. GAT and glycine transporters (GlyTs) are suitable examples, both of which transport 2 Na^+^ and 1 Cl^–^ with one molecule of Glu and gamma-aminobutyric acid (GABA) or glycine, respectively (Zhou and Danbolt, 2013). The transmembrane transport of Glu by astrocytes is mediated by SNAT3/5, co-transport 1 Na^+^ and reverse proton transport (Deitmer et al., 2003; Uwechue et al., 2012). Interestingly, in addition to the efflux of Na^+^ via these above transporters. Excessive intracellular Na^+^ can also be improved by Na^+^/Ca2^+^/Li^+^ exchanger (NCLX) absorbing Na^+^ into mitochondria (Palty et al., 2010). This suggests that mitochondria can act as Na^+^ storage, which is proved by the fact that the Na^+^ concentration of mitochondria in astrocytes is higher than that of surrounding cytoplasm (Ben-Kasus Nissim et al., 2017; Meyer et al., 2019). However, it is interesting to note that reverse operation of the NCX can also lead to Ca^2+^ overload and subsequently cell death (Boscia et al., 2016; Song et al., 2020). Therefore, not only do we need to understand the ionic gradients of transporter ions and the membrane potential of astrocytes to make clear the reversal mechanisms, but the corresponding ion overload caused by reversal is also a matter of concern.

During cerebral ischemia, the efflux of K^+^ from the cell increases due to damage to the cell membrane, which increases the extracellular K^+^ concentration, resulting in neuronal hyperexcitability and apoptosis (van Putten et al., 2021). Astrocytes can sense the increase of extracellular K^+^ concentration and regulate K^+^ through NKA uptake (Hertz et al., 2015; Larsen et al., 2016). In addition, K^+^ uptake by astrocytes involves Kir4.1 channel (Steinhäuser et al., 2012). Kir4.1 is inwardly rectifying the K^+^ channel, which is only expressed in glial cells and the highest in astrocytes (Olsen et al., 2015). In addition, the sodium-potassium-2 chloride-cotransporter 1 (NKCC1) can also mediate the K^+^ regulation of astrocytes when the K^+^ concentration outside the cells increases strongly. After uptake of K^+^ by astrocytes, they are released to the low K^+^ region through gap junction to buffer the K^+^ concentration level of extracellular fluid and restore the K^+^ concentration of extracellular fluid to a stable level. This process is also known as K^+^ spatial buffering (Kofuji and Newman, 2004). However, it should be noted that the long-term activation of NKCC1 will lead to a considerable accumulation of K^+^, Na^+^, and Cl^–^, which will cause the swelling of astrocytes (Su et al., 2002; Lenart et al., 2004).

#### The Dual Role of Glial Scar

Under normal physiological conditions, astrocytes can isolate and protect the CNS (Iliff et al., 2012; Nedergaard, 2013). After ischemic stroke, reactive astrocytes will migrate to the injury site. With the accumulation of time, a large number of reactive astrocytes gather at the edge of injury, and then combine with glycoprotein to form glial scar. The proliferation of reactive astrocytes and the formation of glial scar are considered to be related to fibroblast growth factor (FGF), epidermal growth factor (EGF), adenosine triphosphate (ATP), and endothelin-1 (Levison et al., 2000; Gadea et al., 2008; Neary and Zimmermann, 2009). The environment of neurons is full of a large number of toxic factors after ischemic stroke, such as ion overload, glutamate, free radicals and a large number of pro-inflammatory factors. Glial scar can isolate the injured part from the healthy tissue, prevent the spread of tissue damage, maintain the balance of ions and liquids in the healthy area, and provide nutritional support for the neurons around the glial scar in the injured area (Faulkner et al., 2004; Rolls et al., 2009; Sofroniew, 2009). Some experiments have shown that glial scar can repair BBB, prevent uncontrolled inflammatory reaction and limit cellular degeneration after injury (Bush et al., 1999). Moreover, a large number of studies have proved the key role of glial scar in nerve repair and protection. For example, the loss of scar can lead to the spread of inflammation (Burda and Sofroniew, 2014; Sofroniew, 2014), increase of injury area, more severe demyelination and neuron loss. Similarly, the recovery of clinical function is also limited (Myer et al., 2006). In addition, reactive astrocytes can secrete some immunomodulatory factors such as transforming growth factor-β (TGF-β), TNF-α, and proteoglycans such as chondroitin sulfate proteoglycans (CSPGs), thereby directly acting on immune cells (Chung and Benveniste, 1990). CSPG can regulate the migration and activation of dendritic cells, macrophages and other immune cells (Tham et al., 2010).

When ischemia occurs, fibroblasts will be activated along with astrocytes and constantly migrate to the injured area. A large number of fibroblasts gathered and began to proliferate and secrete ECMs such as collagen and fibronectin protein grafting, etc. In the late stage of ischemia, fibroblasts and extracellular matrix secreted by fibroblasts were not wholly removed, nor were they replaced by regenerated tissues. On the contrary, these cells gather in the lesion area and form fibrous scars (fibrous scars mature and coagulate over time but persist for a long time), the glial component of which is composed of reactive astrocytes (Fitch and Silver, 2008). Many experiments have shown that the formation of fibrous scars is an important reason to hinder axon growth (Yiu and He, 2006; Silver et al., 2014). In the late stage of ischemic stroke, the growth of axons will be significantly inhibited. This may be related to three reasons: myelin-related growth inhibitors (He and Koprivica, 2004), chemical repulsion directing molecules and highly sulfated proteoglycans (Liu et al., 2006)—especially CSPG, which are components of the ECM.

## Conclusion and Prospect

So far, most of the treatment strategies for ischemic stroke are aimed at neurons, and no significant progress has been made. Astrocytes are the most widely distributed cells in mammalian brain and the largest cells in glial cells, which are closely involved in all processes of ischemic stroke, although most functions are still controversial. Interestingly, astrocytes also have phagocytic function, which has been considered as the characteristic function of microglia (Fourgeaud et al., 2016). Reactive astrocyte proliferation is one of the classic pathological features of ischemic stroke, which has both advantages and disadvantages. We can reasonably speculate that evolution has chosen such an acute response to deal with the body damage, which ensures the repair of the damaged body at a certain cost. Notably, astrocytes have a dual role in ischemic stroke, where it participates in multiple emergency and repair processes in different ways. Some astrocyte derived factors have multiple or diametrically opposite properties. For example, astrocyte derived BDNF can promote neuronal survival under physiological conditions, but the abnormal up regulation of BDNF and its corresponding receptor Tyrosine Kinase receptor B will lead to excessive NO production and therefore mediates neurotoxicity (Linnerbauer et al., 2020). Glial scar can isolate the injured part from the healthy tissue, but the later glial scar will aggravate inflammation, inhibit axon growth and hinder the recovery of motor function. In view of the duality of astrocyte function and disease progression has a great relationship, inhibiting or promoting the proliferation of reactive astrocytes is clearly not a sensible therapeutic strategy. By contrast, we should first explore and clarify the timing and mechanism of astrocytes participating in ischemic stroke and then purposefully inhibit or promote the expression of some genes or factors derived of reactive astrocytes in specific stage. This could be a promising direction for targeted therapeutic strategies for astrocytes. However, the limitation of this strategy is that all aspects of astrocyte involvement are usually interactive, suggesting that any attempt to interfere with negative effects may also affect protective function. Therefore, the specific mechanism and time window of astrocytes need to be further explored.

## Author Contributions

MY and Y-SG designed the structure of the manuscript. YH and Z-KG managed the literature searches and analyses. X-YS wrote the manuscript. XB assisted with the improvement of the manuscript. All authors contributed to the article and approved the final manuscript.

## Conflict of Interest

The authors declare that the research was conducted in the absence of any commercial or financial relationships that could be construed as a potential conflict of interest.

## Publisher’s Note

All claims expressed in this article are solely those of the authors and do not necessarily represent those of their affiliated organizations, or those of the publisher, the editors and the reviewers. Any product that may be evaluated in this article, or claim that may be made by its manufacturer, is not guaranteed or endorsed by the publisher.

## References

[B1] Abbott N. J. (2002). Astrocyte-endothelial interactions and blood-brain barrier permeability. *J. Anat.* 200 629–638. 10.1046/j.1469-7580.2002.00064.x 12162730 PMC1570746

[B2] Abbott N. J. Rönnbäck L. Hansson E. (2006). Astrocyte-endothelial interactions at the blood-brain barrier. *Nat. Rev. Neurosci.* 7 41–53. 10.1038/nrn1824 16371949

[B3] Abudara V. Retamal M. A. CityplaceDel Rio R. Orellana J. A. (2018). Synaptic functions of hemichannels and pannexons: a double-edged sword. *Front. Mol. Neurosci.* 11:435. 10.3389/fnmol.2018.00435 30564096 PMC6288452

[B4] Al-Ahmady Z. S. (2018). Selective drug delivery approaches to lesioned brain through blood brain barrier disruption. *Expert Opin. Drug Deliv.* 15 335–349. 10.1080/17425247.2018.1444601 29466890

[B5] Anderson C. M. Swanson R. A. (2000). Astrocyte glutamate transport: review of properties, regulation, and physiological functions. *Glia* 32 1–14. 10975906

[B6] András placeI. E. Deli M. A. Veszelka S. Hayashi K. Hennig B. Toborek M. (2007). The NMDA and AMPA/KA receptors are involved in glutamate-induced alterations of occludin expression and phosphorylation in brain endothelial cells. *J. Cereb. Blood Flow Metab.* 27 1431–1443. 10.1038/sj.jcbfm.9600445 17245419

[B7] Arba F. Leigh R. Inzitari D. Warach S. J. Luby M. Lees K. R. (2017). Blood-brain barrier leakage increases with small vessel disease in acute ischemic stroke. *Neurology* 89 2143–2150. 10.1212/wnl.0000000000004677 29070665 PMC5696647

[B8] Argaw A. T. Asp L. Zhang J. Navrazhina K. Pham T. Mariani J. N. (2012). Astrocyte-derived VEGF-A drives blood-brain barrier disruption in CNS inflammatory disease. *J. Clin. Invest.* 122 2454–2468. 10.1172/jci60842 22653056 PMC3386814

[B9] Argaw A. T. Gurfein B. T. Zhang Y. Zameer A. John G. R. (2009). VEGF-mediated disruption of endothelial CLN-5 promotes blood-brain barrier breakdown. *Proc. Natl. Acad. Sci. U S A.* 106 1977–1982. 10.1073/pnas.0808698106 19174516 PMC2644149

[B10] Asahi M. Wang X. Mori T. Sumii T. Jung J. C. Moskowitz M. A. (2001). Effects of matrix metalloproteinase-9 gene knock-out on the proteolysis of blood-brain barrier and white matter components after cerebral ischemia. *J. Neurosci.* 21 7724–7732. 10.1523/jneurosci.21-19-07724.2001 11567062 PMC6762894

[B11] Attwell D. Buchan A. M. Charpak S. Lauritzen M. Macvicar B. A. Newman E. A. (2010). Glial and neuronal control of brain blood flow. *Nature* 468 232–243. 10.1038/nature09613 21068832 PMC3206737

[B12] Bake S. Okoreeh A. Khosravian H. Sohrabji F. (2019). Insulin-like Growth Factor (IGF)-1 treatment stabilizes the microvascular cytoskeleton under ischemic conditions. *Exp. Neurol.* 311 162–172. 10.1016/j.expneurol.2018.09.016 30287160 PMC6263796

[B13] Balasingam V. Tejada-Berges T. Wright E. Bouckova R. Yong V. W. (1994). Reactive astrogliosis in the neonatal mouse brain and its modulation by cytokines. *J. Neurosci.* 14 846–856. 10.1523/jneurosci.14-02-00846.1994 8301364 PMC6576825

[B14] Ben-Kasus Nissim T. Zhang X. Elazar A. Roy S. Stolwijk J. A. Zhou Y. (2017). Mitochondria control store-operated Ca(2+) entry through Na(+) and redox signals. *EMBO J.* 36 797–815. 10.15252/embj.201592481 28219928 PMC5350565

[B15] Bernaudin M. Nedelec A. S. Divoux D. MacKenzie E. T. Petit E. Schumann-Bard P. (2002). Normobaric hypoxia induces tolerance to focal permanent cerebral ischemia in association with an increased expression of hypoxia-inducible factor-1 and its target genes, erythropoietin and VEGF, in the adult mouse brain. *J. Cereb. Blood Flow Metab.* 22 393–403. 10.1097/00004647-200204000-200204003 11919510

[B16] Boscia F. Begum G. Pignataro G. Sirabella R. Cuomo O. Casamassa A. (2016). Glial Na(+) -dependent ion transporters in pathophysiological conditions. *Glia* 64 1677–1697. 10.1002/glia.23030 27458821 PMC5238576

[B17] Brines M. (2002). What evidence supports use of erythropoietin as a novel neurotherapeutic? *Oncology* 16(9 Suppl. 10) 79–89. 12380958

[B18] Budi E. H. Duan D. Derynck R. (2017). Transforming growth Factor-β receptors and smads: regulatory complexity and functional versatility. *Trends Cell Biol.* 27 658–672. 10.1016/j.tcb.2017.04.005 28552280

[B19] Buemi M. Cavallaro E. Floccari F. Sturiale A. Aloisi C. Trimarchi M. (2003). The pleiotropic effects of erythropoietin in the central nervous system. *J. Neuropathol. Exp. Neurol.* 62 228–236. 10.1093/jnen/62.3.228 12638727

[B20] Buffo A. Rolando C. Ceruti S. (2010). Astrocytes in the damaged brain: molecular and cellular insights into their reactive response and healing potential. *Biochem. Pharmacol.* 79 77–89. 10.1016/j.bcp.2009.09.014 19765548

[B21] Buffo A. Vosko M. R. Ertürk D. Hamann G. F. Jucker M. Rowitch D. (2005). Expression pattern of the transcription factor Olig2 in response to brain injuries: implications for neuronal repair. *Proc. Natl. Acad. Sci. U S A.* 102 18183–18188. 10.1073/pnas.0506535102 16330768 PMC1312388

[B22] Burda J. E. Sofroniew M. V. (2014). Reactive gliosis and the multicellular response to CNS damage and disease. *Neuron* 81 229–248. 10.1016/j.neuron.2013.12.034 24462092 PMC3984950

[B23] Bush T. G. Puvanachandra N. Horner C. H. Polito A. Ostenfeld T. Svendsen C. N. (1999). Leukocyte infiltration, neuronal degeneration, and neurite outgrowth after ablation of scar-forming, reactive astrocytes in adult transgenic mice. *Neuron* 23 297–308. 10.1016/s0896-6273(00)80781-80783 10399936

[B24] Cai J. Chen Y. Cai W. H. Hurlock E. C. Wu H. Kernie S. G. (2007). A crucial role for Olig2 in white matter astrocyte development. *Development* 134 1887–1899. 10.1242/dev.02847 17428828

[B25] Camacho A. Massieu L. (2006). Role of glutamate transporters in the clearance and release of glutamate during ischemia and its relation to neuronal death. *Arch. Med. Res.* 37 11–18. 10.1016/j.arcmed.2005.05.014 16314180

[B26] Ceyzériat K. Abjean L. Carrillo-de Sauvage M. A. Ben Haim L. Escartin C. (2016). The complex STATes of astrocyte reactivity: how are they controlled by the JAK-STAT3 pathway? *Neuroscience* 330 205–218. 10.1016/j.neuroscience.2016.05.043 27241943

[B27] Chen Y. Miles D. K. Hoang T. Shi J. Hurlock E. Kernie S. G. (2008). The basic helix-loop-helix transcription factor olig2 is critical for reactive astrocyte proliferation after cortical injury. *J. Neurosci.* 28 10983–10989. 10.1523/jneurosci.3545-08.2008 18945906 PMC2631240

[B28] Chen Y. Vartiainen N. E. Ying W. Chan P. H. Koistinaho J. Swanson R. A. (2001). Astrocytes protect neurons from nitric oxide toxicity by a glutathione-dependent mechanism. *J. Neurochem.* 77 1601–1610. 10.1046/j.1471-4159.2001.00374.x 11413243

[B29] Cheng L. Zhao H. Zhang W. Liu B. Liu Y. Guo Y. (2013). Overexpression of conserved dopamine neurotrophic factor (CDNF) in astrocytes alleviates endoplasmic reticulum stress-induced cell damage and inflammatory cytokine secretion. *Biochem. Biophys. Res. Commun.* 435 34–39. 10.1016/j.bbrc.2013.04.029 23624196

[B30] Cheng X. Yeung P. K. K. Zhong K. Zilundu P. L. M. Zhou L. Chung S. K. (2019). Astrocytic endothelin-1 overexpression promotes neural progenitor cells proliferation and differentiation into astrocytes via the Jak2/Stat3 pathway after stroke. *J. Neuroinflamm.* 16:227. 10.1186/s12974-019-1597-y 31733648 PMC6858703

[B31] Cheng Z. J. Dai T. M. Shen Y. Y. He J. L. Li J. Tu J. L. (2018). Atorvastatin pretreatment attenuates ischemic brain edema by suppressing aquaporin 4. *J. Stroke Cerebrovasc. Dis.* 27 3247–3255. 10.1016/j.jstrokecerebrovasdis.2018.07.011 30093197

[B32] Chesler M. (2003). Regulation and modulation of pH in the brain. *Physiol. Rev.* 83 1183–1221. 10.1152/physrev.00010.2003 14506304

[B33] Choi D. W. (1988). Glutamate neurotoxicity and diseases of the nervous system. *Neuron* 1 623–634. 10.1016/0896-6273(88)90162-90166 2908446

[B34] Chung placeI. Y. Benveniste E. N. (1990). Tumor necrosis factor-alpha production by astrocytes. induction by lipopolysaccharide, IFN-gamma, and IL-1 beta. *J. Immunol.* 144 2999–3007. 2109008

[B35] Clément T. Rodriguez-Grande B. Badaut J. (2020). Aquaporins in brain edema. *J. Neurosci. Res.* 98 9–18. 10.1002/jnr.24354 30430614

[B36] Cohen E. Dillin A. (2008). The insulin paradox: aging, proteotoxicity and neurodegeneration. *Nat. Rev. Neurosci.* 9 759–767. 10.1038/nrn2474 18769445 PMC2692886

[B37] CityplaceColombo E. Cordiglieri C. Melli G. Newcombe J. Krumbholz M. Parada L. F. (2012). Stimulation of the neurotrophin receptor TrkB on astrocytes drives nitric oxide production and neurodegeneration. *J. Exp. Med.* 209 521–535. 10.1084/jem.20110698 22393127 PMC3302220

[B38] CityplaceColombo E. Farina C. (2016). Astrocytes: key regulators of neuroinflammation. *Trends Immunol.* 37 608–620. 10.1016/j.it.2016.06.006 27443914

[B39] Da T. Verkman A. S. (2004). Aquaporin-4 gene disruption in mice protects against impaired retinal function and cell death after ischemia. *Invest. Ophthalmol. Vis. Sci.* 45 4477–4483. 10.1167/iovs.04-0940 15557457

[B40] Dagonnier M. Donnan G. A. CityplaceDavis S. M. Dewey H. M. Howells D. W. (2021). Acute stroke biomarkers: are we there yet? *Front. Neurol.* 12:619721. 10.3389/fneur.2021.619721 33633673 PMC7902038

[B41] Deitmer J. W. Bröer A. Bröer S. (2003). Glutamine efflux from astrocytes is mediated by multiple pathways. *J. Neurochem.* 87 127–135. 10.1046/j.1471-4159.2003.01981.x 12969260

[B42] del Zoppo G. J. (2009). Inflammation and the neurovascular unit in the setting of focal cerebral ischemia. *Neuroscience* 158 972–982. 10.1016/j.neuroscience.2008.08.028 18824084 PMC2665879

[B43] Di Giovanni S. Movsesyan V. Ahmed F. Cernak placeI. Schinelli S. Stoica B. (2005). Cell cycle inhibition provides neuroprotection and reduces glial proliferation and scar formation after traumatic brain injury. *Proc. Natl. Acad. Sci. U S A.* 102 8333–8338. 10.1073/pnas.0500989102 15923260 PMC1149422

[B44] Dringen R. (2000). Metabolism and functions of glutathione in brain. *Prog. Neurobiol.* 62 649–671. 10.1016/s0301-0082(99)00060-x 10880854

[B45] Dringen R. Brandmann M. Hohnholt M. C. Blumrich E. M. (2015). Glutathione-Dependent Detoxification Processes in Astrocytes. *Neurochem. Res.* 40 2570–2582. 10.1007/s11064-014-1481-1481 25428182

[B46] Dromard C. Bartolami S. Deleyrolle L. Takebayashi H. Ripoll C. Simonneau L. (2007). NG2 and Olig2 expression provides evidence for phenotypic deregulation of cultured central nervous system and peripheral nervous system neural precursor cells. *Stem Cells* 25 340–353. 10.1634/stemcells.2005-2556 17053213

[B47] Duan S. CityplaceAnderson C. M. Keung E. C. Chen Y. Chen Y. Swanson R. A. (2003). P2X7 receptor-mediated release of excitatory amino acids from astrocytes. *J. Neurosci.* 23 1320–1328. 10.1523/jneurosci.23-04-01320.2003 12598620 PMC6742264

[B48] Eroglu C. Barres B. A. (2010). Regulation of synaptic connectivity by glia. *Nature* 468 223–231. 10.1038/nature09612 21068831 PMC4431554

[B49] Fandrey J. (2004). Oxygen-dependent and tissue-specific regulation of erythropoietin gene expression. *Am. J. Physiol. Regul. Integr. Comp. Physiol.* 286 R977–R988. 10.1152/ajpregu.00577.2003 15142852

[B50] Faulkner J. R. Herrmann J. E. Woo M. J. Tansey K. E. Doan N. B. Sofroniew M. V. (2004). Reactive astrocytes protect tissue and preserve function after spinal cord injury. *J. Neurosci.* 24 2143–2155. 10.1523/jneurosci.3547-03.2004 14999065 PMC6730429

[B51] Feigin V. L. Krishnamurthi R. V. Parmar P. Norrving B. Mensah G. A. Bennett D. A. (2015). Update on the Global Burden of Ischemic and Hemorrhagic Stroke in 1990-2013: the GBD 2013 Study. *Neuroepidemiology* 45 161–176. 10.1159/000441085 26505981 PMC4633282

[B52] Fitch M. T. Silver J. (2008). CNS injury, glial scars, and inflammation: inhibitory extracellular matrices and regeneration failure. *Exp. Neurol.* 209 294–301. 10.1016/j.expneurol.2007.05.014 17617407 PMC2268907

[B53] Fourgeaud L. Través P. G. Tufail Y. Leal-Bailey H. Lew E. D. Burrola P. G. (2016). TAM receptors regulate multiple features of microglial physiology. *Nature* 532 240–244. 10.1038/nature17630 27049947 PMC5358512

[B54] Franke H. Grosche J. Schädlich H. Krügel U. Allgaier C. Illes P. (2001). P2X receptor expression on astrocytes in the nucleus accumbens of rats. *Neuroscience* 108 421–429. 10.1016/s0306-4522(01)00416-x 11738256

[B55] Fu S. Gu Y. Jiang J. Q. Chen X. Xu M. Chen X. (2014). Calycosin-7-O-β-D-glucoside regulates nitric oxide /caveolin-1/matrix metalloproteinases pathway and protects blood-brain barrier integrity in experimental cerebral ischemia-reperfusion injury. *J. Ethnopharmacol.* 155 692–701. 10.1016/j.jep.2014.06.015 24930357

[B56] Fukuda S. Abematsu M. Mori H. Yanagisawa M. Kagawa T. Nakashima K. (2007). Potentiation of astrogliogenesis by STAT3-mediated activation of bone morphogenetic protein-Smad signaling in neural stem cells. *Mol. Cell. Biol.* 27 4931–4937. 10.1128/mcb.02435-2436 17452461 PMC1951480

[B57] Gabay L. Lowell S. Rubin L. L. Anderson D. J. (2003). Deregulation of dorsoventral patterning by FGF confers trilineage differentiation capacity on CNS stem cells in vitro. *Neuron* 40 485–499. 10.1016/s0896-6273(03)00637-638 14642274

[B58] Gadea A. Schinelli S. Gallo V. (2008). Endothelin-1 regulates astrocyte proliferation and reactive gliosis via a JNK/c-Jun signaling pathway. *J. Neurosci.* 28 2394–2408. 10.1523/jneurosci.5652-07.2008 18322086 PMC2695974

[B59] Gerkau N. J. Rakers C. Petzold G. C. Rose C. R. (2017). Differential effects of energy deprivation on intracellular sodium homeostasis in neurons and astrocytes. *J. Neurosci. Res.* 95 2275–2285. 10.1002/jnr.23995 28150887

[B60] CityplaceGriffin S. Clark J. B. Canevari L. (2005). Astrocyte-neurone communication following oxygen-glucose deprivation. *J. Neurochem.* 95 1015–1022. 10.1111/j.1471-4159.2005.03418.x 16190880

[B61] Gu Y. Zheng G. Xu M. Li Y. Chen X. Zhu W. (2012). Caveolin-1 regulates nitric oxide-mediated matrix metalloproteinases activity and blood-brain barrier permeability in focal cerebral ischemia and reperfusion injury. *J. Neurochem.* 120 147–156. 10.1111/j.1471-4159.2011.07542.x 22007835

[B62] Guo X. Jiang Q. Tuccitto A. Chan D. Alqawlaq S. Won G. J. (2018). The AMPK-PGC-1α signaling axis regulates the astrocyte glutathione system to protect against oxidative and metabolic injury. *Neurobiol. Dis.* 113 59–69. 10.1016/j.nbd.2018.02.004 29438738

[B63] Hao J. Q. He X. Y. Yang X. Xiao Y. C. Duan S. Q. Wang H. (2021). Acetazolamide Alleviate Cerebral Edema Induced by Ischemic Stroke Through Inhibiting the Expression of AQP4 mRNA. *Neurocrit. Care* 10.1007/s12028-021-01261-w Online ahead of print. 34302276

[B64] He Z. Koprivica V. (2004). The Nogo signaling pathway for regeneration block. *Annu. Rev. Neurosci.* 27 341–368. 10.1146/annurev.neuro.27.070203.144340 15217336

[B65] Hertz L. Song D. Xu J. Peng L. Gibbs M. E. (2015). Role of the Astrocytic Na(+), K(+)-ATPase in K(+) Homeostasis in Brain: K(+) Uptake, Signaling Pathways and Substrate Utilization. *Neurochem. Res.* 40 2505–2516. 10.1007/s11064-014-1505-x 25555706

[B66] Hill S. A. Fu M. Garcia A. D. R. (2021). Sonic hedgehog signaling in astrocytes. *Cell Mol. Life. Sci.* 78 1393–1403. 10.1007/s00018-020-03668-3668 33079226 PMC7904711

[B67] Ho J. D. Yeh R. Sandstrom A. Chorny I. Harries W. E. Robbins R. A. (2009). CityplaceCrystal structure of human aquaporin 4 at 1.8 *A and its mechanism of conductance*. *Proc. Natl. Acad. Sci. U S A.* 106 7437–7442. 10.1073/pnas.0902725106 19383790 PMC2678640

[B68] Hossmann K. A. (2012). The two pathophysiologies of focal brain ischemia: implications for translational stroke research. *J. Cereb. Blood Flow Metab.* 32 1310–1316. 10.1038/jcbfm.2011.186 22234335 PMC3390813

[B69] Huang Y. H. Bergles D. E. (2004). Glutamate transporters bring competition to the synapse. *Curr. Opin. Neurobiol.* 14 346–352. 10.1016/j.conb.2004.05.007 15194115

[B70] Hung V. K. Yeung P. K. Lai A. K. Ho M. C. Lo A. C. Chan K. C. (2015). Selective astrocytic endothelin-1 overexpression contributes to dementia associated with ischemic stroke by exaggerating astrocyte-derived amyloid secretion. *J. Cereb. Blood Flow Metab.* 35 1687–1696. 10.1038/jcbfm.2015.109 26104290 PMC4640314

[B71] Igarashi Y. Utsumi H. CityplaceChiba H. Yamada-Sasamori Y. Tobioka H. Kamimura Y. (1999). Glial cell line-derived neurotrophic factor induces barrier function of endothelial cells forming the blood-brain barrier. *Biochem. Biophys. Res. Commun.* 261 108–112. 10.1006/bbrc.1999.0992 10405331

[B72] Iliff J. J. Wang M. Liao Y. Plogg B. A. Peng W. Gundersen G. A. (2012). A paravascular pathway facilitates CSF flow through the brain parenchyma and the clearance of interstitial solutes, including amyloid β. *Sci. Transl. Med.* 4:147ra111. 10.1126/scitranslmed.3003748 22896675 PMC3551275

[B73] Jia C. Keasey M. P. Lovins C. Hagg T. (2018). Inhibition of astrocyte FAK-JNK signaling promotes subventricular zone neurogenesis through CNTF. *Glia* 66 2456–2469. 10.1002/glia.23498 30500112 PMC6863602

[B74] Jiang S. Xia R. Jiang Y. Wang L. Gao F. (2014a). Vascular endothelial growth factors enhance the permeability of the mouse blood-brain barrier. *PLoS One* 9:e86407. 10.1371/journal.pone.0086407 24551038 PMC3925082

[B75] Jiang Z. Li C. Arrick D. M. Yang S. Baluna A. E. Sun H. (2014b). Role of nitric oxide synthases in early blood-brain barrier disruption following transient focal cerebral ischemia. *PLoS One* 9:e93134. 10.1371/journal.pone.0093134 24671193 PMC3966853

[B76] Justicia C. Gabriel C. Planas A. M. (2000). Activation of the JAK/STAT pathway following transient focal cerebral ischemia: signaling through Jak1 and Stat3 in astrocytes. *Glia* 30 253–270. 10756075 10.1002/(sici)1098-1136(200005)30:3<253::aid-glia5>3.0.co;2-o

[B77] Kajihara H. Tsutsumi E. Kinoshita A. Nakano J. Takagi K. Takeo S. (2001). Activated astrocytes with glycogen accumulation in ischemic penumbra during the early stage of brain infarction: immunohistochemical and electron microscopic studies. *Brain Res.* 909 92–101. 10.1016/s0006-8993(01)02640-2643 11478925

[B78] Kang S. S. Keasey M. P. placeCityArnold S. A. Reid R. Geralds J. Hagg T. (2013). Endogenous CNTF mediates stroke-induced adult CNS neurogenesis in mice. *Neurobiol. Dis.* 49 68–78. 10.1016/j.nbd.2012.08.020 22960105 PMC3657597

[B79] Kimelberg H. K. Goderie S. K. Higman S. Pang S. Waniewski R. A. (1990). Swelling-induced release of glutamate, aspartate, and taurine from astrocyte cultures. *J. Neurosci.* 10 1583–1591. 10.1523/jneurosci.10-05-01583.1990 1970603 PMC6570070

[B80] Klein M. A. Möller J. C. Jones L. L. Bluethmann H. Kreutzberg G. W. Raivich G. (1997). Impaired neuroglial activation in interleukin-6 deficient mice. *Glia* 19 227–233. 9063729 10.1002/(sici)1098-1136(199703)19:3<227::aid-glia5>3.0.co;2-w

[B81] Kofuji P. Newman E. A. (2004). Potassium buffering in the central nervous system. *Neuroscience* 129 1045–1056. 10.1016/j.neuroscience.2004.06.008 15561419 PMC2322935

[B82] Kostandy B. B. (2012). The role of glutamate in neuronal ischemic injury: the role of spark in fire. *Neurol. Sci.* 33 223–237. 10.1007/s10072-011-0828-825 22044990

[B83] Koyama Y. (2014). Signaling molecules regulating phenotypic conversions of astrocytes and glial scar formation in damaged nerve tissues. *Neurochem. Int.* 78 35–42. 10.1016/j.neuint.2014.08.005 25180676

[B84] Koyama Y. (2021). Endothelin ET(B) Receptor-Mediated Astrocytic Activation: pathological Roles in Brain Disorders. *Int. J. Mol. Sci.* 22:4333. 10.3390/ijms22094333 33919338 PMC8122402

[B85] Koyama Y. Michinaga S. (2012). Regulations of astrocytic functions by endothelins: roles in the pathophysiological responses of damaged brains. *J. Pharmacol. Sci.* 118 401–407. 10.1254/jphs.11r13cp 22447302

[B86] Kuboyama K. Harada H. Tozaki-Saitoh H. Tsuda M. Ushijima K. Inoue K. (2011). Astrocytic P2Y(1) receptor is involved in the regulation of cytokine/chemokine transcription and cerebral damage in a rat model of cerebral ischemia. *J. Cereb. Blood Flow Metab.* 31 1930–1941. 10.1038/jcbfm.2011.49 21487414 PMC3185880

[B87] Larpthaveesarp A. Georgevits M. Ferriero D. M. Gonzalez F. F. (2016). Delayed erythropoietin therapy improves histological and behavioral outcomes after transient neonatal stroke. *Neurobiol. Dis.* 93 57–63. 10.1016/j.nbd.2016.04.006 27142685 PMC4930700

[B88] Larsen B. R. Stoica A. MacAulay N. (2016). Managing brain extracellular K(+) during neuronal activity: the physiological role of the Na(+)/K(+)-ATPase subunit isoforms. *Front. Physiol.* 7:141. 10.3389/fphys.2016.00141 27148079 PMC4841311

[B89] Lee H. U. Yamazaki Y. Tanaka K. F. Furuya K. Sokabe M. Hida H. (2013). Increased astrocytic ATP release results in enhanced excitability of the hippocampus. *Glia* 61 210–224. 10.1002/glia.22427 23018918

[B90] Lee S. W. Kim W. J. Choi Y. K. Song H. S. Son M. J. Gelman I. H. (2003). SSeCKS regulates angiogenesis and tight junction formation in blood-brain barrier. *Nat. Med.* 9 900–906. 10.1038/nm889 12808449

[B91] Lee T. H. Kato H. Kogure K. Itoyama Y. (1996). Temporal profile of nerve growth factor-like immunoreactivity after transient focal cerebral ischemia in rats. *Brain Res.* 713 199–210. 10.1016/0006-8993(95)01510-1518 8724992

[B92] Lenart B. Kintner D. B. Shull G. E. Sun D. (2004). Na-K-Cl cotransporter-mediated intracellular Na+ accumulation affects Ca2+ signaling in astrocytes in an in vitro ischemic model. *J. Neurosci.* 24 9585–9597. 10.1523/jneurosci.2569-04.2004 15509746 PMC6730155

[B93] Leonova J. Thorlin T. Aberg N. D. Eriksson P. S. Rönnbäck L. Hansson E. (2001). Endothelin-1 decreases glutamate uptake in primary cultured rat astrocytes. *Am. J. Physiol. Cell Physiol.* 281 C1495–C1503. 10.1152/ajpcell.2001.281.5.C1495 11600412

[B94] Levison S. W. Jiang F. J. Stoltzfus O. K. Ducceschi M. H. (2000). IL-6-type cytokines enhance epidermal growth factor-stimulated astrocyte proliferation. *Glia* 32 328–337. 11102972 10.1002/1098-1136(200012)32:3<328::aid-glia110>3.0.co;2-7

[B95] Li H. Zhang N. Lin H. Y. Yu Y. Cai Q. Y. Ma L. (2014). Histological, cellular and behavioral assessments of stroke outcomes after photothrombosis-induced ischemia in adult mice. *BMC Neurosci.* 15:58. 10.1186/1471-2202-15-58 24886391 PMC4039545

[B96] Li S. Gu X. Yi S. (2017). The Regulatory Effects of Transforming Growth Factor-β on Nerve Regeneration. *Cell Transplant.* 26 381–394. 10.3727/096368916x693824 27983926 PMC5657701

[B97] Liddelow S. A. Guttenplan K. A. Clarke L. E. Bennett F. C. Bohlen C. J. Schirmer L. (2017). Neurotoxic reactive astrocytes are induced by activated microglia. *Nature* 541 481–487. 10.1038/nature21029 28099414 PMC5404890

[B98] Liebner S. Czupalla C. J. Wolburg H. (2011). Current concepts of blood-brain barrier development. *Int. J. Dev. Biol.* 55 467–476. 10.1387/ijdb.103224sl 21769778

[B99] Linnerbauer M. Wheeler M. A. Quintana F. J. (2020). Astrocyte Crosstalk in CNS Inflammation. *Neuron* 108 608–622. 10.1016/j.neuron.2020.08.012 32898475 PMC7704785

[B100] Lipton P. (1999). Ischemic cell death in brain neurons. *Physiol. Rev.* 79 1431–1568. 10.1152/physrev.1999.79.4.1431 10508238

[B101] Liu B. P. Cafferty W. B. Budel S. O. Strittmatter S. M. (2006). Extracellular regulators of axonal growth in the adult central nervous system. *Philos. Trans. R. Soc. Lond. B Biol. Sci.* 361 1593–1610. 10.1098/rstb.2006.1891 16939977 PMC1664666

[B102] Liu L. Zhao B. Xiong X. Xia Z. (2018). The neuroprotective roles of sonic hedgehog signaling pathway in ischemic stroke. *Neurochem. Res.* 43 2199–2211. 10.1007/s11064-018-2645-2641 30267379

[B103] Liu X. Hunter C. Weiss H. R. Chi O. Z. (2010). Effects of blockade of ionotropic glutamate receptors on blood-brain barrier disruption in focal cerebral ischemia. *Neurol. Sci.* 31 699–703. 10.1007/s10072-010-0241-245 20217443

[B104] Liu Z. Chopp M. (2016). Astrocytes, therapeutic targets for neuroprotection and neurorestoration in ischemic stroke. *Prog. Neurobiol.* 144 103–120. 10.1016/j.pneurobio.2015.09.008 26455456 PMC4826643

[B105] Liu Z. Li Y. Cui Y. Roberts C. Lu M. Wilhelmsson U. (2014). Beneficial effects of gfap/vimentin reactive astrocytes for axonal remodeling and motor behavioral recovery in mice after stroke. *Glia* 62 2022–2033. 10.1002/glia.22723 25043249 PMC4307923

[B106] Lo A. C. Chen A. Y. Hung V. K. Yaw L. P. Fung M. K. Ho M. C. (2005). Endothelin-1 overexpression leads to further water accumulation and brain edema after middle cerebral artery occlusion via aquaporin 4 expression in astrocytic end-feet. *J. Cereb. Blood Flow Metab.* 25 998–1011. 10.1038/sj.jcbfm.9600108 15815585

[B107] CityLogan A. placeStateBerry M. Gonzalez A. M. Frautschy S. A. Sporn M. B. Baird A. (1994). Effects of transforming growth factor beta 1 on scar production in the injured central nervous system of the rat. *Eur. J. Neurosci.* 6 355–363. 10.1111/j.1460-9568.1994.tb00278.x 8019673

[B108] Longuemare M. C. Rose C. R. Farrell K. Ransom B. R. Waxman S. G. Swanson R. A. (1999). K(+)-induced reversal of astrocyte glutamate uptake is limited by compensatory changes in intracellular Na+. *Neuroscience* 93 285–292. 10.1016/s0306-4522(99)00152-159 10430492

[B109] Longuemare M. C. Swanson R. A. (1995). Excitatory amino acid release from astrocytes during energy failure by reversal of sodium-dependent uptake. *J. Neurosci. Res.* 40 379–386. 10.1002/jnr.490400312 7745632

[B110] Lu L. Hogan-Cann A. D. Globa A. K. Lu P. Nagy J. I. Bamji S. X. (2019). Astrocytes drive cortical vasodilatory signaling by activating endothelial NMDA receptors. *J. Cereb. Blood Flow Metab.* 39 481–496. 10.1177/0271678x17734100 29072857 PMC6421257

[B111] Magnus T. Coksaygan T. Korn T. Xue H. Arumugam T. V. Mughal M. R. (2007). Evidence that nucleocytoplasmic Olig2 translocation mediates brain-injury-induced differentiation of glial precursors to astrocytes. *J. Neurosci. Res.* 85 2126–2137. 10.1002/jnr.21368 17510983

[B112] Malarkey E. B. Parpura V. (2008). Mechanisms of glutamate release from astrocytes. *Neurochem. Int.* 52 142–154. 10.1016/j.neuint.2007.06.005 17669556 PMC2267911

[B113] Mallet R. T. Ryou M. G. (2017). Erythropoietin: endogenous protection of ischemic brain. *Vitam Horm.* 105 197–232. 10.1016/bs.vh.2017.01.002 28629519

[B114] Marais A. D. (2019). Apolipoprotein E in lipoprotein metabolism, health and cardiovascular disease. *Pathology* 51 165–176. 10.1016/j.pathol.2018.11.002 30598326

[B115] Marshall C. A. Novitch B. G. Goldman J. E. (2005). Olig2 directs astrocyte and oligodendrocyte formation in postnatal subventricular zone cells. *J. Neurosci.* 25 7289–7298. 10.1523/jneurosci.1924-05.2005 16093378 PMC6725308

[B116] Mason R. B. Pluta R. M. Walbridge S. Wink D. A. Oldfield E. H. Boock R. J. (2000). Production of reactive oxygen species after reperfusion in vitro and in vivo: protective effect of nitric oxide. *J. Neurosurg.* 93 99–107. 10.3171/jns.2000.93.1.0099 10883911

[B117] McLeod middlenameplaceD. middlenameD. Parsons M. W. Hood R. Hiles B. Allen J. McCann S. K. (2015). Perfusion computed tomography thresholds defining ischemic penumbra and infarct core: studies in a rat stroke model. *Int. J. Stroke* 10 553–559. 10.1111/ijs.12147 24138577

[B118] Meyer J. Untiet V. Fahlke C. Gensch T. Rose C. R. (2019). Quantitative determination of cellular [Na(+)] by fluorescence lifetime imaging with CoroNaGreen. *J. Gen. Physiol.* 151 1319–1331. 10.1085/jgp.201912404 31597684 PMC6829561

[B119] Miao Y. Qiu Y. Lin Y. Miao Z. Zhang J. Lu X. (2011). Protection by pyruvate against glutamate neurotoxicity is mediated by astrocytes through a glutathione-dependent mechanism. *Mol. Biol. Rep.* 38 3235–3242. 10.1007/s11033-010-9998-9990 20182801

[B120] Michinaga S. Ishida A. Takeuchi R. Koyama Y. (2013). Endothelin-1 stimulates cyclin D1 expression in rat cultured astrocytes via activation of Sp1. *Neurochem. Int.* 63 25–34. 10.1016/j.neuint.2013.04.004 23619396

[B121] Mizee M. R. Wooldrik D. Lakeman K. A. van het Hof B. Drexhage J. A. Geerts D. (2013). Retinoic acid induces blood-brain barrier development. *J. Neurosci.* 33 1660–1671. 10.1523/jneurosci.1338-12.2013 23345238 PMC6618717

[B122] Mizui T. Kinouchi H. Chan P. H. (1992). Depletion of brain glutathione by buthionine sulfoximine enhances cerebral ischemic injury in rats. *Am. J. Physiol.* 262(2 Pt 2), H313–H317. 10.1152/ajpheart.1992.262.2.H313 1539690

[B123] Moon L. D. Fawcett J. W. (2001). Reduction in CNS scar formation without concomitant increase in axon regeneration following treatment of adult rat brain with a combination of antibodies to TGFbeta1 and beta2. *Eur. J. Neurosci.* 14 1667–1677. 10.1046/j.0953-816x.2001.01795.x 11860461

[B124] Morikawa M. Fryer J. D. Sullivan P. M. Christopher E. A. Wahrle S. E. DeMattos R. B. (2005). Production and characterization of astrocyte-derived human apolipoprotein E isoforms from immortalized astrocytes and their interactions with amyloid-beta. *Neurobiol. Dis.* 19 66–76. 10.1016/j.nbd.2004.11.005 15837562

[B125] Mulligan S. J. MacVicar B. A. (2004). Calcium transients in astrocyte endfeet cause cerebrovascular constrictions. *Nature* 431 195–199. 10.1038/nature02827 15356633

[B126] Myer D. J. Gurkoff G. G. Lee S. M. Hovda D. A. Sofroniew M. V. (2006). Essential protective roles of reactive astrocytes in traumatic brain injury. *Brain* 129(Pt 10) 2761–2772. 10.1093/brain/awl165 16825202

[B127] Nakajima K. Kohsaka S. (2004). Microglia: neuroprotective and neurotrophic cells in the central nervous system. *Curr. Drug. Targets Cardiovasc. Haematol. Disord.* 4 65–84. 10.2174/1568006043481284 15032653

[B128] Nawashiro H. Brenner M. placeCityFukui S. Shima K. Hallenbeck J. M. (2000). High susceptibility to cerebral ischemia in GFAP-null mice. *J. Cereb. Blood Flow Metab.* 20 1040–1044. 10.1097/00004647-200007000-200007003 10908037

[B129] Neary J. T. Kang Y. Tran M. Feld J. (2005). Traumatic injury activates protein kinase B/Akt in cultured astrocytes: role of extracellular ATP and P2 purinergic receptors. *J. Neurotrauma* 22 491–500. 10.1089/neu.2005.22.491 15853465

[B130] Neary J. T. Zimmermann H. (2009). Trophic functions of nucleotides in the central nervous system. *Trends Neurosci.* 32 189–198. 10.1016/j.tins.2009.01.002 19282037

[B131] Nedergaard M. (2013). Neuroscience. garbage truck of the brain. *Science* 340 1529–1530. 10.1126/science.1240514 23812703 PMC3749839

[B132] Nedergaard M. Dirnagl U. (2005). Role of glial cells in cerebral ischemia. *Glia* 50 281–286. 10.1002/glia.20205 15846807

[B133] Nguyen A. Q. Cherry B. H. Scott G. F. Ryou M. G. Mallet R. T. (2014). Erythropoietin: powerful protection of ischemic and post-ischemic brain. *Exp. Biol. Med.* 239 1461–1475. 10.1177/1535370214523703 24595981 PMC4331056

[B134] Obermeier B. Daneman R. Ransohoff R. M. (2013). Development, maintenance and disruption of the blood-brain barrier. *Nat. Med.* 19 1584–1596. 10.1038/nm.3407 24309662 PMC4080800

[B135] O’Callaghan J. P. Kelly K. A. VanGilder R. L. Sofroniew M. V. Miller D. B. (2014). Early activation of STAT3 regulates reactive astrogliosis induced by diverse forms of neurotoxicity. *PLoS One* 9:e102003. 10.1371/journal.pone.0102003 25025494 PMC4098997

[B136] Olsen M. L. Khakh B. S. Skatchkov S. N. Zhou M. Lee C. J. Rouach N. (2015). New insights on astrocyte ion channels: critical for homeostasis and neuron-glia signaling. *J. Neurosci.* 35 13827–13835. 10.1523/jneurosci.2603-15.2015 26468182 PMC4604221

[B137] Ono K. Takebayashi H. Ikenaka K. (2009). Olig2 transcription factor in the developing and injured forebrain; cell lineage and glial development. *Mol. Cells* 27 397–401. 10.1007/s10059-009-0067-62 19390819

[B138] Osei-Owusu J. Yang J. Vitery M. D. C. Qiu Z. (2018). Molecular biology and physiology of Volume-Regulated Anion Channel (VRAC). *Curr. Top. Membr.* 81 177–203. 10.1016/bs.ctm.2018.07.005 30243432 PMC6604840

[B139] Pajarillo E. Rizor A. Lee J. Aschner M. Lee E. (2019). The role of astrocytic glutamate transporters GLT-1 and GLAST in neurological disorders: potential targets for neurotherapeutics. *Neuropharmacology* 161:107559. 10.1016/j.neuropharm.2019.03.002 30851309 PMC6731169

[B140] Pál G. Vincze C. Renner É Wappler E. A. Nagy Z. Lovas G. (2012). Time course, distribution and cell types of induction of transforming growth factor betas following middle cerebral artery occlusion in the rat brain. *PLoS One* 7:e46731. 10.1371/journal.pone.0046731 23056426 PMC3466286

[B141] Palty R. Silverman W. F. Hershfinkel M. Caporale T. Sensi S. L. Parnis J. (2010). NCLX is an essential component of mitochondrial Na+/Ca2+ exchange. *Proc. Natl. Acad. Sci. U S A.* 107 436–441. 10.1073/pnas.0908099107 20018762 PMC2806722

[B142] Panickar K. S. Norenberg M. D. (2005). Astrocytes in cerebral ischemic injury: morphological and general considerations. *Glia* 50 287–298. 10.1002/glia.20181 15846806

[B143] Papadopoulos M. C. Verkman A. S. (2007). Aquaporin-4 and brain edema. *Pediatr. Nephrol.* 22 778–784. 10.1007/s00467-006-0411-410 17347837 PMC6904420

[B144] Parpura V. Scemes E. Spray D. C. (2004). Mechanisms of glutamate release from astrocytes: gap junction “hemichannels”, purinergic receptors and exocytotic release. *Neurochem. Int.* 45 259–264. 10.1016/j.neuint.2003.12.011 15145541

[B145] Pascual O. CityplaceCasper K. B. Kubera C. Zhang J. Revilla-Sanchez R. Sul J. Y. (2005). Astrocytic purinergic signaling coordinates synaptic networks. *Science* 310 113–116. 10.1126/science.1116916 16210541

[B146] Pitt J. Wilcox K. C. Tortelli V. Diniz L. P. Oliveira M. S. Dobbins C. (2017). Neuroprotective astrocyte-derived insulin/insulin-like growth factor 1 stimulates endocytic processing and extracellular release of neuron-bound Aβ oligomers. *Mol. Biol. Cell* 28 2623–2636. 10.1091/mbc.E17-06-0416 28963439 PMC5620371

[B147] Pow D. V. (2001). Visualising the activity of the cystine-glutamate antiporter in glial cells using antibodies to aminoadipic acid, a selectively transported substrate. *Glia* 34 27–38. 10.1002/glia.1037 11284017

[B148] Qu W. S. Wang Y. H. Wang J. P. Tang Y. X. Zhang Q. Tian D. S. (2010). Galectin-1 enhances astrocytic BDNF production and improves functional outcome in rats following ischemia. *Neurochem. Res.* 35 1716–1724. 10.1007/s11064-010-0234-z 20689988

[B149] Rabchevsky A. G. Weinitz J. M. Coulpier M. Fages C. Tinel M. Junier M. P. (1998). A role for transforming growth factor alpha as an inducer of astrogliosis. *J. Neurosci.* 18 10541–10552. 10.1523/jneurosci.18-24-10541.1998 9852591 PMC6793335

[B150] Ridet J. L. Malhotra S. K. Privat A. Gage F. H. (1997). Reactive astrocytes: cellular and molecular cues to biological function. *Trends Neurosci.* 20 570–577. 10.1016/s0166-2236(97)01139-1139 9416670

[B151] Rolls A. Shechter R. Schwartz M. (2009). The bright side of the glial scar in CNS repair. *Nat. Rev. Neurosci.* 10 235–241. 10.1038/nrn2591 19229242

[B152] Rose C. R. Ransom B. R. (1996). Intracellular sodium homeostasis in rat hippocampal astrocytes. *J. Physiol.* 491(Pt 2) 291–305. 10.1113/jphysiol.1996.sp021216 8866855 PMC1158726

[B153] Rose C. R. Waxman S. G. Ransom B. R. (1998). Effects of glucose deprivation, chemical hypoxia, and simulated ischemia on Na+ homeostasis in rat spinal cord astrocytes. *J. Neurosci.* 18 3554–3562. 10.1523/jneurosci.18-10-03554.1998 9570787 PMC6793162

[B154] Rose C. R. Ziemens D. Verkhratsky A. (2020). On the special role of NCX in astrocytes: translating Na(+)-transients into intracellular Ca(2+) signals. *Cell Calcium* 86:102154. 10.1016/j.ceca.2019.102154 31901681

[B155] Rosenberg P. A. Aizenman E. (1989). Hundred-fold increase in neuronal vulnerability to glutamate toxicity in astrocyte-poor cultures of rat cerebral cortex. *Neurosci. Lett.* 103 162–168. 10.1016/0304-3940(89)90569-90567 2570387

[B156] Rossi D. J. Oshima T. Attwell D. (2000). Glutamate release in severe brain ischaemia is mainly by reversed uptake. *Nature* 403 316–321. 10.1038/35002090 10659851

[B157] Ruscher K. Freyer D. Karsch M. Isaev N. Megow D. Sawitzki B. (2002). Erythropoietin is a paracrine mediator of ischemic tolerance in the brain: evidence from an in vitro model. *J. Neurosci.* 22 10291–10301. 10.1523/jneurosci.22-23-10291.2002 12451129 PMC6758760

[B158] Ruscher K. Kuric E. Wieloch T. (2012). Levodopa treatment improves functional recovery after experimental stroke. *Stroke* 43 507–513. 10.1161/strokeaha.111.638767 22096034

[B159] Ryu H. Lee J. Zaman K. Kubilis J. Ferrante R. J. Ross B. D. (2003). Sp1 and Sp3 are oxidative stress-inducible, antideath transcription factors in cortical neurons. *J. Neurosci.* 23 3597–3606. 10.1523/jneurosci.23-09-03597.2003 12736330 PMC6742168

[B160] Sahlender D. A. Savtchouk placeI. Volterra A. (2014). What do we know about gliotransmitter release from astrocytes? *Philos. Trans. R. Soc. Lond. B Biol. Sci.* 369:20130592. 10.1098/rstb.2013.0592 25225086 PMC4173278

[B161] Sasaki R. (2003). Pleiotropic functions of erythropoietin. *Int. Med.* 42 142–149. 10.2169/internalmedicine.42.142 12636232

[B162] Sato M. (2006). Upregulation of the Wnt/beta-catenin pathway induced by transforming growth factor-beta in hypertrophic scars and keloids. *Acta Derm. Venereol.* 86 300–307. 10.2340/00015555-15101 16874413

[B163] Senn R. Elkind M. S. Montaner J. Christ-Crain M. Katan M. (2014). Potential role of blood biomarkers in the management of nontraumatic intracerebral hemorrhage. *Cerebrovasc. Dis.* 38 395–409. 10.1159/000366470 25471997

[B164] Sharp C. D. Hines I. Houghton J. CityWarren A. CityplaceJackson T. H. T. Jawahar A. (2003). Glutamate causes a loss in human cerebral endothelial barrier integrity through activation of NMDA receptor. *Am. J. Physiol. Heart Circ. Physiol.* 285 H2592–H2598. 10.1152/ajpheart.00520.2003 12893641

[B165] Shen Y. Sun A. Wang Y. Cha D. Wang H. Wang F. (2012). Upregulation of mesencephalic astrocyte-derived neurotrophic factor in glial cells is associated with ischemia-induced glial activation. *J. Neuroinflamm.* 9:254. 10.1186/1742-2094-9-254 23173607 PMC3576245

[B166] Shi Z. F. Fang Q. Chen Y. Xu L. X. Wu M. Jia M. (2021). Methylene blue ameliorates brain edema in rats with experimental ischemic stroke via inhibiting aquaporin 4 expression. *Acta Pharmacol. Sin.* 42 382–392. 10.1038/s41401-020-0468-465 32665706 PMC8027449

[B167] Silver J. Schwab M. E. Popovich P. G. (2014). Central nervous system regenerative failure: role of oligodendrocytes, astrocytes, and microglia. *Cold Spring Harb. Perspect. Biol.* 7:a020602. 10.1101/cshperspect.a020602 25475091 PMC4355267

[B168] Simard J. M. country-regionplaceKent T. A. Chen M. Tarasov K. V. Gerzanich V. (2007). Brain oedema in focal ischaemia: molecular pathophysiology and theoretical implications. *Lancet Neurol.* 6 258–268. 10.1016/s1474-4422(07)70055-70058 17303532 PMC2725365

[B169] Sirén A. L. Fratelli M. Brines M. Goemans C. Casagrande S. Lewczuk P. (2001). Erythropoietin prevents neuronal apoptosis after cerebral ischemia and metabolic stress. *Proc. Natl. Acad. Sci. U S A.* 98 4044–4049. 10.1073/pnas.051606598 11259643 PMC31176

[B170] Sofroniew M. V. (2009). Molecular dissection of reactive astrogliosis and glial scar formation. *Trends Neurosci.* 32 638–647. 10.1016/j.tins.2009.08.002 19782411 PMC2787735

[B171] Sofroniew M. V. (2014). Multiple roles for astrocytes as effectors of cytokines and inflammatory mediators. *Neuroscientist* 20 160–172. 10.1177/1073858413504466 24106265

[B172] Sofroniew M. V. (2015). Astrocyte barriers to neurotoxic inflammation. *Nat. Rev. Neurosci.* 16 249–263. 10.1038/nrn3898 25891508 PMC5253239

[B173] Sofroniew M. V. Vinters H. V. (2010). Astrocytes: biology and pathology. *Acta Neuropathol.* 119 7–35. 10.1007/s00401-009-0619-618 20012068 PMC2799634

[B174] Song S. Luo L. Sun B. Sun D. (2020). Roles of glial ion transporters in brain diseases. *Glia* 68 472–494. 10.1002/glia.23699 31418931 PMC6957693

[B175] Song Y. Pimentel C. Walters K. Boller L. Ghiasvand S. Liu J. (2016). Neuroprotective levels of IGF-1 exacerbate epileptogenesis after brain injury. *Sci. Rep.* 6:32095. 10.1038/srep32095 27561791 PMC4999804

[B176] Spector R. (2016). Dehydroascorbic acid for the treatment of acute ischemic stroke. *Med. Hypotheses* 89 32–36. 10.1016/j.mehy.2016.01.021 26968905

[B177] Spray D. C. Ye Z. C. Ransom B. R. (2006). Functional connexin “hemichannels”: a critical appraisal. *Glia* 54 758–773. 10.1002/glia.20429 17006904

[B178] Sriram K. Benkovic S. A. Hebert M. A. Miller D. B. O’Callaghan J. P. (2004). Induction of gp130-related cytokines and activation of JAK2/STAT3 pathway in astrocytes precedes up-regulation of glial fibrillary acidic protein in the 1-methyl-4-phenyl-1,2,3,6-tetrahydropyridine model of neurodegeneration: key signaling pathway for astrogliosis in vivo? *J. Biol. Chem.* 279 19936–19947. 10.1074/jbc.M309304200 14996842

[B179] Stapf C. Mohr J. P. (2002). Ischemic stroke therapy. *Annu. Rev. Med.* 53 453–475. 10.1146/annurev.med.53.082901.104106 11818485

[B180] Steinhäuser C. Seifert G. Bedner P. (2012). Astrocyte dysfunction in temporal lobe epilepsy: K+ channels and gap junction coupling. *Glia* 60 1192–1202. 10.1002/glia.22313 22328245

[B181] Stoica A. Larsen B. R. Assentoft M. Holm R. Holt L. M. Vilhardt F. (2017). The α2β2 isoform combination dominates the astrocytic Na(+) /K(+) -ATPase activity and is rendered nonfunctional by the α2.G301R familial hemiplegic migraine type 2-associated mutation. *Glia* 65 1777–1793. 10.1002/glia.23194 28787093 PMC12247536

[B182] Stokum J. A. placeKurland D. B. Gerzanich V. Simard J. M. (2015). Mechanisms of astrocyte-mediated cerebral edema. *Neurochem. Res.* 40 317–328. 10.1007/s11064-014-1374-1373 24996934 PMC4284155

[B183] Su G. Kintner D. B. Flagella M. Shull G. E. Sun D. (2002). Astrocytes from Na(+)-K(+)-Cl(-) cotransporter-null mice exhibit absence of swelling and decrease in EAA release. *Am. J. Physiol. Cell Physiol.* 282 C1147–C1160. 10.1152/ajpcell.00538.2001 11940530

[B184] Sun Q. Guo S. Wang C. C. Sun X. Wang D. Xu N. (2015). Cross-talk between TGF-β/Smad pathway and Wnt/β-catenin pathway in pathological scar formation. *Int. J. Clin. Exp. Pathol.* 8 7631–7639. 26261683 PMC4526017

[B185] Susarla B. T. Laing E. D. Yu P. Katagiri Y. Geller H. M. Symes A. J. (2011). Smad proteins differentially regulate transforming growth factor-β-mediated induction of chondroitin sulfate proteoglycans. *J. Neurochem.* 119 868–878. 10.1111/j.1471-4159.2011.07470.x 21895657 PMC3197872

[B186] Swanson R. A. Ying W. Kauppinen T. M. (2004). Astrocyte influences on ischemic neuronal death. *Curr. Mol. Med.* 4 193–205. 10.2174/1566524043479185 15032713

[B187] Szatkowski M. Attwell D. (1994). Triggering and execution of neuronal death in brain ischaemia: two phases of glutamate release by different mechanisms. *Trends Neurosci.* 17 359–365. 10.1016/0166-2236(94)90040-x 7529438

[B188] Takahashi S. (2021). Neuroprotective function of high glycolytic activity in astrocytes: common roles in stroke and neurodegenerative diseases. *Int. J. Mol. Sci.* 22:6568. 10.3390/ijms22126568 34207355 PMC8234992

[B189] Takahashi S. Izawa Y. Suzuki N. (2012). Astroglial pentose phosphate pathway rates in response to high-glucose environments. *ASN Neuro* 4:e00078. 10.1042/an20120002 22300409 PMC3310304

[B190] Tanaka J. Toku K. Zhang B. Ishihara K. Sakanaka M. Maeda N. (1999). Astrocytes prevent neuronal death induced by reactive oxygen and nitrogen species. *Glia* 28 85–96. 10533053 10.1002/(sici)1098-1136(199911)28:2<85::aid-glia1>3.0.co;2-y

[B191] Tatsumi K. Takebayashi H. Manabe T. Tanaka K. F. Makinodan M. Yamauchi T. (2008). Genetic fate mapping of Olig2 progenitors in the injured adult cerebral cortex reveals preferential differentiation into astrocytes. *J. Neurosci. Res.* 86 3494–3502. 10.1002/jnr.21862 18816798

[B192] Tham M. Ramasamy S. Gan H. T. Ramachandran A. Poonepalli A. Yu Y. H. (2010). CSPG is a secreted factor that stimulates neural stem cell survival possibly by enhanced EGFR signaling. *PLoS One* 5:e15341. 10.1371/journal.pone.0015341 21179491 PMC3001889

[B193] Tokita Y. Keino H. Matsui F. Aono S. Ishiguro H. Higashiyama S. (2001). Regulation of neuregulin expression in the injured rat brain and cultured astrocytes. *J. Neurosci.* 21 1257–1264. 10.1523/jneurosci.21-04-01257.2001 11160396 PMC6762219

[B194] Toral-Rios D. Patiño-López G. Gómez-Lira G. Gutiérrez R. Becerril-Pérez F. Rosales-Córdova A. (2020). Activation of STAT3 regulates reactive astrogliosis and neuronal death induced by AβO neurotoxicity. *Int. J. Mol. Sci.* 21:7458. 10.3390/ijms21207458 33050466 PMC7590075

[B195] Ullensvang K. Lehre K. P. Storm-Mathisen J. Danbolt N. C. (1997). Differential developmental expression of the two rat brain glutamate transporter proteins GLAST and GLT. *Eur. J. Neurosci.* 9 1646–1655. 10.1111/j.1460-9568.1997.tb01522.x 9283819

[B196] Ullian E. M. Christopherson K. S. Barres B. A. (2004). Role for glia in synaptogenesis. *Glia* 47 209–216. 10.1002/glia.20082 15252809

[B197] Ullian E. M. Sapperstein S. K. Christopherson K. S. Barres B. A. (2001). Control of synapse number by glia. *Science* 291 657–661. 10.1126/science.291.5504.657 11158678

[B198] Uwechue N. M. Marx M. C. Chevy Q. Billups B. (2012). Activation of glutamate transport evokes rapid glutamine release from perisynaptic astrocytes. *J. Physiol.* 590 2317–2331. 10.1113/jphysiol.2011.226605 22411007 PMC3424755

[B199] van Putten M. Fahlke C. Kafitz K. W. Hofmeijer J. Rose C. R. (2021). Dysregulation of astrocyte ion homeostasis and its relevance for stroke-induced brain damage. *Int. J. Mol. Sci.* 22:5679. 10.3390/ijms22115679 34073593 PMC8198632

[B200] Verkhratsky A. Trebak M. Perocchi F. Khananshvili D. Sekler placeI. (2018). Crosslink between calcium and sodium signalling. *Exp. Physiol.* 103 157–169. 10.1113/ep086534 29210126 PMC6813793

[B201] Verkman A. S. Binder D. K. Bloch O. Auguste K. Papadopoulos M. C. (2006). Three distinct roles of aquaporin-4 in brain function revealed by knockout mice. *Biochim. Biophys. Acta* 1758 1085–1093. 10.1016/j.bbamem.2006.02.018 16564496

[B202] Vidale S. Agostoni E. (2017). Endovascular treatment of ischemic stroke: an updated meta-analysis of efficacy and safety. *Vasc. Endovasc. Surg.* 51 215–219. 10.1177/1538574417698905 28424039

[B203] Wang J. Sareddy G. R. Lu Y. Pratap U. P. Tang F. Greene K. M. (2020). Astrocyte-Derived estrogen regulates reactive astrogliosis and is neuroprotective following ischemic brain injury. *J. Neurosci.* 40 9751–9771. 10.1523/jneurosci.0888-20.2020 33158962 PMC7726540

[B204] Wang X. F. Cynader M. S. (2000). Astrocytes provide cysteine to neurons by releasing glutathione. *J. Neurochem.* 74 1434–1442. 10.1046/j.1471-4159.2000.0741434.x 10737599

[B205] Washburn K. B. Neary J. T. (2006). P2 purinergic receptors signal to STAT3 in astrocytes: difference in STAT3 responses to P2Y and P2X receptor activation. *Neuroscience* 142 411–423. 10.1016/j.neuroscience.2006.06.034 16905269

[B206] Wevers N. R. de Vries H. E. (2016). Morphogens and blood-brain barrier function in health and disease. *Tissue Barriers* 4:e1090524. 10.1080/21688370.2015.1090524 27141417 PMC4836462

[B207] Winter C. G. Saotome Y. Levison S. W. Hirsh D. (1995). A role for ciliary neurotrophic factor as an inducer of reactive gliosis, the glial response to central nervous system injury. *Proc. Natl. Acad. Sci. U S A.* 92 5865–5869. 10.1073/pnas.92.13.5865 7597043 PMC41602

[B208] Wrigley S. Arafa D. Tropea D. (2017). Insulin-Like growth factor 1: at the crossroads of brain development and aging. *Front. Cell Neurosci.* 11:14. 10.3389/fncel.2017.00014 28203146 PMC5285390

[B209] Xia X. G. Hofmann H. D. Deller T. Kirsch M. (2002). Induction of STAT3 signaling in activated astrocytes and sprouting septal neurons following entorhinal cortex lesion in adult rats. *Mol. Cell. Neurosci.* 21 379–392. 10.1006/mcne.2002.1180 12498781

[B210] Xia Y. P. He Q. W. Li Y. N. Chen S. C. Huang M. Wang Y. (2013). Recombinant human sonic hedgehog protein regulates the expression of ZO-1 and occludin by activating angiopoietin-1 in stroke damage. *PLoS One* 8:e68891. 10.1371/journal.pone.0068891 23894369 PMC3720889

[B211] Xiao Q. Du Y. Wu W. Yip H. K. (2010). Bone morphogenetic proteins mediate cellular response and, together with Noggin, regulate astrocyte differentiation after spinal cord injury. *Exp. Neurol.* 221 353–366. 10.1016/j.expneurol.2009.12.003 20005873

[B212] Xiao W. Wang W. Chen W. Sun L. Li X. Zhang C. (2014). GDNF is involved in the barrier-inducing effect of enteric glial cells on intestinal epithelial cells under acute ischemia reperfusion stimulation. *Mol. Neurobiol.* 50 274–289. 10.1007/s12035-014-8730-8739 24878766

[B213] Xing L. Yang T. Cui S. Chen G. (2019). connexin hemichannels in astrocytes: role in CNS disorders. *Front. Mol. Neurosci.* 12:23. 10.3389/fnmol.2019.00023 30787868 PMC6372977

[B214] Yan M. Dai H. Ding T. Dai A. Zhang F. Yu L. (2011). Effects of dexmedetomidine on the release of glial cell line-derived neurotrophic factor from rat astrocyte cells. *Neurochem. Int.* 58 549–557. 10.1016/j.neuint.2011.01.013 21241763

[B215] Yang Y. Estrada E. Y. Thompson J. F. Liu W. Rosenberg G. A. (2007). Matrix metalloproteinase-mediated disruption of tight junction proteins in cerebral vessels is reversed by synthetic matrix metalloproteinase inhibitor in focal ischemia in rat. *J. Cereb. Blood Flow Metab.* 27 697–709. 10.1038/sj.jcbfm.9600375 16850029

[B216] Yang Y. Rosenberg G. A. (2011). MMP-mediated disruption of claudin-5 in the blood-brain barrier of rat brain after cerebral ischemia. *Methods Mol. Biol.* 762 333–345. 10.1007/978-1-61779-185-7_24 21717368 PMC4950933

[B217] Ye Z. C. Wyeth M. S. Baltan-Tekkok S. Ransom B. R. (2003). Functional hemichannels in astrocytes: a novel mechanism of glutamate release. *J. Neurosci.* 23 3588–3596. 10.1523/jneurosci.23-09-03588.2003 12736329 PMC6742182

[B218] Yiu G. He Z. (2006). Glial inhibition of CNS axon regeneration. *Nat. Rev. Neurosci.* 7 617–627. 10.1038/nrn1956 16858390 PMC2693386

[B219] Yu H. Wang P. An P. Xue Y. (2012). Recombinant human angiopoietin-1 ameliorates the expressions of ZO-1, occludin, VE-cadherin, and PKCα signaling after focal cerebral ischemia/reperfusion in rats. *J. Mol. Neurosci.* 46 236–247. 10.1007/s12031-011-9584-9585 21710361

[B220] Zeng X. N. Xie L. L. Liang R. Sun X. L. Fan Y. Hu G. (2012). AQP4 knockout aggravates ischemia/reperfusion injury in mice. *CNS Neurosci. Ther.* 18 388–394. 10.1111/j.1755-5949.2012.00308.x 22533723 PMC6493383

[B221] Zhang S. An Q. Wang T. Gao S. Zhou G. (2018). Autophagy- and MMP-2/9-mediated reduction and redistribution of ZO-1 Contribute to hyperglycemia-increased blood-brain barrier permeability during early reperfusion in stroke. *Neuroscience* 377 126–137. 10.1016/j.neuroscience.2018.02.035 29524637

[B222] Zhang T. Wang X. F. Wang Z. C. Lou D. Fang Q. Q. Hu Y. Y. (2020). Current potential therapeutic strategies targeting the TGF-β/Smad signaling pathway to attenuate keloid and hypertrophic scar formation. *Biomed. Pharmacother.* 129:110287. 10.1016/j.biopha.2020.110287 32540643

[B223] Zhang Y. Xu K. Liu Y. Erokwu B. O. Zhao P. Flask C. A. (2019). Increased cerebral vascularization and decreased water exchange across the blood-brain barrier in aquaporin-4 knockout mice. *PLoS One* 14:e0218415. 10.1371/journal.pone.0218415 31220136 PMC6586297

[B224] Zhang Z. G. Zhang L. Jiang Q. Zhang R. Davies K. Powers C. (2000). VEGF enhances angiogenesis and promotes blood-brain barrier leakage in the ischemic brain. *J. Clin. Invest.* 106 829–838. 10.1172/jci9369 11018070 PMC517814

[B225] Zhao H. Liu Y. Cheng L. Liu B. Zhang W. Guo Y. J. (2013). Mesencephalic astrocyte-derived neurotrophic factor inhibits oxygen-glucose deprivation-induced cell damage and inflammation by suppressing endoplasmic reticulum stress in rat primary astrocytes. *J. Mol. Neurosci.* 51 671–678. 10.1007/s12031-013-0042-44 23760988

[B226] Zhao H. Wang R. Wu X. Liang J. Qi Z. Liu X. (2015). Erythropoietin delivered via intra-arterial infusion reduces endoplasmic reticulum stress in brain microvessels of rats following cerebral ischemia and reperfusion. *J. Neuroimmune Pharmacol.* 10 153–161. 10.1007/s11481-014-9571-z 25626440

[B227] Zhou Y. Danbolt N. C. (2013). GABA and glutamate transporters in brain. *Front. Endocrinol.* 4:165. 10.3389/fendo.2013.00165 24273530 PMC3822327

[B228] Zhu Z. Zhang Q. Yu Z. Zhang L. Tian D. Zhu S. (2007). Inhibiting cell cycle progression reduces reactive astrogliosis initiated by scratch injury in vitro and by cerebral ischemia in vivo. *Glia* 55 546–558. 10.1002/glia.20476 17243097

[B229] Zonta M. Angulo M. C. Gobbo S. Rosengarten B. Hossmann K. A. Pozzan T. (2003). Neuron-to-astrocyte signaling is central to the dynamic control of brain microcirculation. *Nat. Neurosci.* 6 43–50. 10.1038/nn980 12469126

